# The relationship of publication language, study population, risk of bias, and treatment effects in acupuncture related systematic reviews: a meta-epidemiologic study

**DOI:** 10.1186/s12874-023-01904-w

**Published:** 2023-04-20

**Authors:** Jing Li, Xu Hui, Liang Yao, Anya Shi, Peijing Yan, Yuan Yao, Qi Wang, Yanfang Ma, Dang Wei, Lei Lan, Lingxiao Chen, Lijiao Yan, Fang Fang, Huijuan Li, Xiaowen Feng, Jingxi Wu, Yifan Qiao, Wenhao Zhang, Janne Esill, Chengdong Qiao, Kehu Yang

**Affiliations:** 1 Health technology Assessment Centre, Evidence Based Social Science Research Center, School of Public Health, Lanzhou University, Lanzhou, China; 2 Evidence Based Medicine Center, School of Basic Medical Sciences, Lanzhou University, Lanzhou, China; 3 Health Research Methodology, Department of Health Research Methods, Evidence and Impact, McMaster University, Hamilton, ON Canada; 4 Second Clinical Medical College of Lanzhou University, Lanzhou, China; 5 West China school of public health, Sichuan university, Chengdu, China; 6 First Clinical Medical College of Lanzhou University, Lanzhou, China; 7 Health Policy PhD Program and McMaster Health Forum, McMaster University, Hamilton, ON Canada; 8 School of Chinese Medicine, Hong Kong Chinese Medicine Clinical Study Center, Hong Kong Baptist University, Hong Kong, China; 9 Chinese EQUATOR Centre, Hong Kong, China; 10 Institute of Environmental Medicine, Karolinska Institute, Stockholm, Sweden; 11 School of Acupuncture, Moxibustion and Massage, Chengdu University of Traditional Chinese Medicine, Chengdu, China; 12 Faculty of Medicine and Health, The Back Pain Research Team, Sydney Musculoskeletal Health, The Kolling Institute, University of Sydney, Sydney, NSW Australia; 13 Centre for Evidence-Based Chinese Medicine, Beijing University of Chinese Medicine, Beijing, China; 14 Guangzhou University of Chinese Medicine, Guangzhou, China; 15 Institute of Global Health, University of Geneva, Geneva, Sweden; 16 The First Hospital of Lanzhou University, Lanzhou, China; 17 Key Laboratory of Evidence Based Medicine and Knowledge Translation of Gansu Province, Lanzhou, China

**Keywords:** Systematic reviews, Acupuncture, Meta-epidemiologic study, Risk of bias, Publication language

## Abstract

**Background:**

There are debates in acupuncture related systematic reviews and meta-analyses on whether searching Chinese databases to get more Chinese-language studies may increase the risk of bias and overestimate the effect size, and whether the treatment effects of acupuncture differ between Chinese and non-Chinese populations.

**Methods:**

In this meta-epidemiological study, we searched the Cochrane library from its inception until December 2021, and identified systematic reviews and meta-analyses with acupuncture as one of the interventions. Paired reviewers independently screened the reviews and extracted the information. We repeated the meta-analysis of the selected outcomes to separately pool the results of Chinese- and non-Chinese-language acupuncture studies and presented the pooled estimates as odds ratios (OR) with 95% confidence interval (CI). We calculated the Ratio of ORs (ROR) by dividing the OR of the Chinese-language trials by the OR of the non-Chinese-language trials, and the ROR by dividing the OR of trials addressing Chinese population by the OR of trials addressing non-Chinese population. We explored whether the impact of a high risk of bias on the effect size differed between studies published in Chinese- and in non-Chinese-language, and whether the treatment effects of acupuncture differed between Chinese and non-Chinese population.

**Results:**

We identified 84 Cochrane acupuncture reviews involving 33 Cochrane groups, of which 31 reviews (37%) searched Chinese databases. Searching versus not searching Chinese databases significantly increased the contribution of Chinese-language literature both to the total number of included trials (54% vs. 15%) and the sample size (40% vs. 15%). When compared with non-Chinese-language trials, Chinese-language trials were associated with a larger effect size (pooled ROR 0.51, 95% CI 0.29 to 0.91). We also observed a higher risk of bias in Chinese-language trials in blinding of participants and personnel (97% vs. 51%) and blinding of outcome assessment (93% vs. 47%). The higher risk of bias was associated with a larger effect estimate in both Chinese-language (allocation concealment: high/unclear risk vs. low risk, ROR 0.43, 95% CI 0.21 to 0.87) and non-Chinese-language studies (blinding of participants and personnel: high/unclear risk vs. low risk, ROR 0.41, 95% CI 0.23 to 0.74). However, we found no evidence that the higher risk of bias would increase the effect size of acupuncture in Chinese-language studies more often than in non-Chinese-language studies (the confidence intervals of all ROR in the high-risk group included 1, Table 3). We further found acupuncture appeared to be more effective in Chinese than in non-Chinese population (Table 4).

**Conclusions:**

The findings of this study suggest the higher risk of bias may lead to an overestimation of the treatment effects of acupuncture but would not increase the treatment effects in Chinese-language studies more often than in other language studies. The difference in treatment effects of acupuncture was probably associated with differences in population characteristics.

**Trial registration:**

We registered our protocol on the Open Science Framework (OSF) (10.17605/OSF.IO/PZ6XR).

**Supplementary Information:**

The online version contains supplementary material available at 10.1186/s12874-023-01904-w.

## Background

Acupuncture is a popular complementary alternative treatment and is widely used both in China and elsewhere, in over 100 countries [1]. Acupuncture can be used to treat various diseases such as chronic pain, urinary incontinence, stroke, arthritis, and insomnia [2–6]. According to the recent collection of articles on acupuncture in the BMJ, over two thousand systematic reviews and meta-analyses (SR/MAs) of acupuncture therapies were published over the past 20 years, and many of them have been used to support recommendations in clinical guidelines [2, 7–9].

Acupuncture is a traditional Chinese form of therapy. The number of publications on acupuncture from Chinese institutions is increasing: according to a survey, studies from China accounted for 47% of the 13,320 acupuncture-related publications indexed in PubMed from 1995 to 2014 [10]. To avoid missing potentially relevant Chinese-language literature on acupuncture, many researchers have begun to search and include acupuncture studies in Chinese in their SR/MAs [11–13]. Based on a prior survey we performed, more than half of Cochrane acupuncture reviews have included Chinese-language studies, and many of them also searched Chinese databases to include more Chinese-language literature [14].

There are however some concerns. First, there have been fears that the inclusion of Chinese-language studies could increase the risk of bias in meta-analyses, and leading to misleading conclusions [15, 16]. Second, given the long application history of acupuncture in China, some people believe that acupuncture might be more effective in the Chinese population than elsewhere [17–19].

However, to our knowledge, no study has so far explored whether the treatment effects of acupuncture differ between Chinese and non-Chinese population, and whether the inclusion of Chinese-language studies would influence the effect size due to the increased risk of bias. To address these questions, we conducted this meta-epidemiologic study. The Cochrane Library has published more than 80 full reviews of acupuncture with rigorous methodology [14], which provided an optimal sample for this study.

## Methods

### Registration and protocol

We registered our protocol on the Open Science Framework (OSF) (10.17605/OSF.IO/PZ6XR). Based on the feedback from the peer reviewers, we conducted additional analyses investigating the differences between populations, which were not included in the original protocol.

### Data sources and selection

We searched the Cochrane Databases of Systematic Reviews from their inception until December 2021 using the keywords “acupuncture”, “needling*”, “acupressur*”, and “electro-acupuncture” (Appendix 1) [20]. Two reviewers (JL and XH) independently screened the studies in two stages: (1) titles and abstracts, and (2) full texts of potentially eligible studies. We included SR/MAs that included acupuncture as one of the interventions. Reviews were excluded if they (1) were withdrawn, (2) did not include any trials, (3) were protocols, or (4) were outdated versions.

### Data extraction

Two reviewers (JL and XH) obtained the following details from each included review using a standardized pre-designed data extraction form: (1) basic information on the review including the first author, year, country, disease, retrieved databases, and language restrictions for searching the literature; (2) information on the included trials such as the number of trials, total sample size, risk of bias (ROB), the language (we accessed the full text of each trial to identify the publication language as Chinese or other), the target population (Chinese or non-Chinese population), and (3) data on binary outcomes including the number of events and sample size. From each review, we selected for analysis one primary binary outcome (as defined by the Cochrane Collaboration) that met the following conditions (applied in the following hierarchical order): (1) the forest plot included both Chinese- and non-Chinese-language studies; (2) the outcome had the largest number of trials; and (3) the outcome had the largest sample size. If no primary outcome met the criteria, we selected a secondary binary outcome according to the same criteria. Disagreements were resolved by discussion.

### Data analysis

We presented the numbers of eligible Chinese-language studies and participants in acupuncture SR/MAs that searched versus did not search Chinese databases. Referring to the previous methodological studies [21–24], we used Review Manager 5.4 to repeat the meta-analyses of the selected outcomes using the same method as reported in the original reviews (fixed or random model, and the application of any correction for zero events) to separately pool the results of Chinese- and non-Chinese-language acupuncture studies, and presented the pooled estimates as odds ratios (OR) with 95% confidence intervals (CI). If necessary, we transformed the direction of the ORs so that OR < 1 was defined as favoring acupuncture.

We then calculated the Ratio of OR (ROR) for each outcome by dividing the OR of the Chinese-language trials by the OR of the non-Chinese-language trials, and the ROR by dividing the OR of trials addressing Chinese populations by the OR of trials addressing non-Chinese populations. To pool overall ROR or ROR in different groups, we used random-effects model, inverse variance method and the metafor package (metagen command) in R 4.1.3 software. If the 95% CI of ROR included 1 (null effect), we concluded that there was not enough evidence neither in favour nor against the Chinese-language acupuncture trials; if the 95% CI was completely below 1, Chinese-language studies were associated with larger effect size than non-Chinese-language studies.

Statistical heterogeneity among studies were measured by the I^2^ statistic and Q test [25]. For the I^2^ test we used the following definitions: [1] 0–40%, heterogeneity not necessarily important; [2] 30–60%, possibly moderate heterogeneity; [3] 50–90%: substantial heterogeneity; and [4] 75–100%: considerable heterogeneity. Subgroup analysis was conducted according to the types of outcomes (primary vs. secondary outcome) and threshold (high/unclear risk vs. low risk) of each ROB domain. We tested subgroup differences and considered P < 0.05 as statistically significant [26].

In each domain of ROB (randomization, concealment, blinding of participants and personnel, blinding of outcome assessment, incomplete outcome data, and selective reporting), we explored whether studies of each domain with the higher risk of bias overestimated the treatment effect of acupuncture in Chinese- and non-Chinese-language studies. ROR less than 1 implies that the treatment effects are overestimated in the trials with the domain with a high risk of bias.

We also explored, in studies with the higher risk of bias (including high and unclear risk of bias), whether the impact of the higher risk of bias on the treatment effect of acupuncture differ between the Chinese- and non-Chinese-language studies. ROR less than 1 implied that the contribution of the higher risk of bias to an overestimation of the treatment effects of acupuncture was greater in Chinese-language than non-Chinese-language studies. Furthermore, we explored whether the treatment effect of acupuncture differed between the Chinese and non-Chinese population. This analysis was restricted to studies with a low risk of bias to avoid confounding from the higher risk of bias. ROR less than 1 implied that acupuncture is more effective in Chinese population than in non-Chinese population.

## Results

### Characteristics of acupuncture reviews

Our study identified 84 Cochrane acupuncture reviews (Appendix Fig. 1, Appendix Table 1) involving 33 Cochrane groups. None of the reviews restricted the language except one [27] that only searched English-language literature. Among the 84 acupuncture reviews, 31 (37%) searched Chinese databases in addition to the standard databases recommended by Cochrane systematic review hanbook, such as MEDLINE, PUBMED, EMBASE, CENTRAL) (see details in Appendix Fig. 3).

As shown in Table 1, 45 acupuncture reviews (28 of which also searched Chinese databases) included studies in both Chinese and in other languages (331/352 = 94% of which were in English). Twenty-one [27–47] of these reviews that included only Chinese-language trials and nine reviews [48–56] that did not include any Chinese-language trials in the forest plots of binary outcomes were excluded from the quantitative analysis (Appendix Table 2). The remaining 15 [20, 57–70] acupuncture reviews included both Chinese- and non-Chinese-language studies in the forest plots of binary outcomes and were included in the quantitative analysis. Appendix Table 2 presents the selected binary outcomes for each review based on the pre-defined criteria (including nine primary and six secondary outcomes).

**Table 1:** The proportion of reviews, Chinese-language trials, and participants in Cochrane acupuncture reviews searching versus not searching Chinese databases

Categories	Searched Chinese databases		Total
	Yes	No	
Review level (n = 84)			
Included at least one Chinese-language trial	28 (90)	17(32)	45
Did not include any Chinese-language trial	3 (10)	36 (68)	39
Total (%)	31 (100)	53 (100)	84
Trial level (n = 852)			
Chinese-language trials	264 (54)	55 (15)	319
Non-Chinese-language trials	228 (46)	305 (85)	533
Total (%)	492 (100)	360 (100)	852
Sample size level (n = 104,225)			
Participants from Chinese-language trials	22,818 (40)	7,154 (15)	29,972
Participants from Non-Chinese-language trials	34,637 (60)	39,616 (85)	74,253
Total (%)	57,455 (100)	46,770 (100)	104,225

Of the 31 reviews that searched Chinese databases, 28 (90%) included Chinese-language studies; of the 53 reviews that did not search Chinese databases, 17 (32%) included Chinese-language studies. The probability of including Chinese-language studies was thus approximately 1.8 times higher if Chinese databases were searched versus if not (90% vs. 32%, Table 1).

The 31 Cochrane acupuncture reviews that searched Chinese databases included a total of 264 Chinese-language trials (54% of all included trials) with 22,818 (40% of all included participants) participants (Table 1). The 53 Cochrane acupuncture reviews that did not search Chinese databases included a total of 55 (15%) Chinese-language trials (identified from standard databases) with 7,154 (15%) participants. Searching versus not searching Chinese databases in the acupuncture reviews, on average, resulted in a 2.6 times higher contribution of Chinese-language studies (54% vs. 15%, Table 1), and 1.7 times higher contribution of participants from Chinese-language studies (40% vs. 15%, Table 1) in the meta-analysis.

### Risk of bias in chinese- and non-chinese-language acupuncture studies

**Fig. 1:**
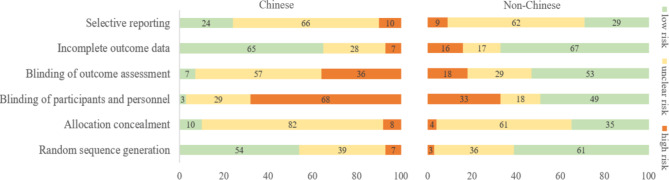
The risk of bias in Chinese- and non-Chinese-language acupuncture trials

The 45 eligible acupuncture reviews included a total of 319 Chinese-language trials (264 from reviews that searched Chinese databases and 55 from reviews that did not search any Chinese databases) and 352 non-Chinese-language trials (191 from reviews that searched Chinese databases and 161 from reviews that did not any search Chinese databases; Appendix Fig. 2). Figure 1 presents the risk of bias in the acupuncture studies by the language of publication, which suggested the Chinese-language acupuncture studies had a higher risk of bias regarding the blinding of participants and personnel (97% vs. 51% having high or unclear risk) and blinding of outcome assessment (93% vs. 47%) than studies written in languages other than Chinese (Fig. 1, Appendix Table 4, Appendix Table 5, Appendix Table 6).

### Overall discrepancies in effect sizes between chinese- and non-chinese-language acupuncture studies

**Fig. 2:**
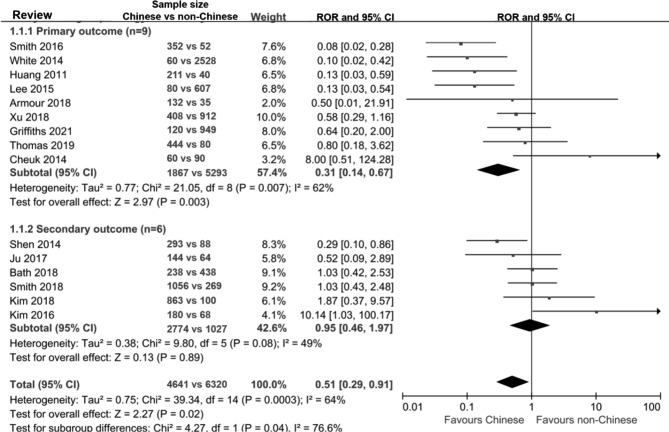
Discrepancies of effect sizes between Chinese- and non-Chinese-language acupuncture trials in the selected binary outcomes

Chinese-language acupuncture studies tended to have a larger effect size than non-Chinese-language studies (pooled ROR 0.51, 95% CI 0.29 to 0.91, Fig. 2 and Appendix Fig. 4).

We found that the higher risk of bias was associated with larger effect size in both Chinese-language studies (allocation concealment: ROR 0.43, 95% CI 0.21 to 0.87, Table 2), and non-Chinese-language studies (blinding of participants and personnel: ROR 0.41, 95% CI 0.23 to 0.74, Table 2).

**Table 2:** Discrepancies of effect sizes of high/unclear risk versus low risk in each risk of bias domain in Chinese- and non-Chinese-language acupuncture trials

Subgroup	No. reviews	No. RCTs (high and unclear risk vs. low risk)	No. sample size (high and unclear risk vs. low risk)	ROR^*^ [95% CI]	p value
Random sequence generation (selection bias)					
Chinese-language trial	8	21 vs. 25	1844 vs. 2551	0.83 [0.44, 1.57]	0.63
Non-Chinese-language trial	3	19 vs. 11	2489 vs. 1084	1.00 [0.66, 1.50]	
Allocation concealment (selection bias)					
Chinese-language trial	3	16 vs. 3	1272 vs. 430	0.43 [0.21, 0.87]*	0.37
Non-Chinese-language trial	4	27 vs. 7	3211 vs. 631	0.65 [0.38, 1.09]	
Blinding of participants and personnel (performance bias)					
Chinese-language trial	0	NA	NA	NA	NA
Non-Chinese-language trial	3	13 vs. 4	2042 vs. 426	0.41 [0.23, 0.74]*	
Blinding of outcome assessment (detection bias)					
Chinese-language trial	2	6 vs. 2	444 vs. 87	0.51 [0.15, 1.78]	0.50
Non-Chinese-language trial	4	8 vs. 13	601 vs. 2305	0.84 [0.40, 1.74]	
Incomplete outcome data (attrition bias)					
Chinese-language trial	5	13 vs. 9	1508 vs. 678	1.78 [0.73, 4.34]	0.52
Non-Chinese-language trial	5	18 vs. 21	2955 vs. 2310	1.24 [0.67, 2.29]	
Selective reporting (reporting bias)					
Chinese-language trial	1	3 vs. 1	262 vs. 294	1.00 [0.29, 3.41]	0.83
Non-Chinese-language trial	3	7 vs. 8	460 vs. 1497	1.18 [0.49, 2.83]	

^*^ ROR less than 1 implies that high risk of bias was associated with a larger effect size than low risk of bias

We found no evidence suggesting that the higher risk of bias would increase the effect size in Chinese-language studies more often than in the non-Chinese-language studies (all confidence intervals of RORs in the higher risk of bias group included 1, Table 3).

**Table 3:** Discrepancies of treatment effects of Chinese- versus non-Chinese-language acupuncture trials stratified by ROB domains with high/unclear risk of bias

ROB domains	No. reviews	No. RCTs (Chinese- vs. non-Chinese-language)	Sample size (Chinese- vs. non-Chinese-language)	ROR^*^ [95% CI]
Random sequence generation (high/unclear risk)	9	20 vs. 26	1524 vs. 3425	0.87 [0.36, 2.11]
Allocation concealment (high/unclear risk)	12	41 vs. 39	3419 vs. 4556	0.60 [0.32, 1.15]
Blinding of participants and personnel (high/unclear risk)	13	51 vs. 23	4509 vs. 2804	0.67 [0.37, 1.21]
Blinding of outcome assessment (high/unclear risk)	13	49 vs. 20	4422 vs. 1452	0.59 [0.29, 1.19]
Incomplete outcome data (high/unclear risk)	4	16 vs. 8	1660 vs. 711	1.26 [0.56, 2.84]
Selective reporting (high/unclear risk)	12	49 vs. 40	4339 vs. 4605	0.52 [0.27, 1.00]

^*^ROR less than 1 implies the high/unclear risk contributed more overestimate to the treatment effects of acupuncture in Chinese-language studies than in non-Chinese-language studies

### Discrepancies in treatment effects between chinese and non-chinese populations

When restricting to low risk of bias studies, we found that acupuncture was associated with larger treatment effects when applied to Chinese than other populations (low risk in random sequence generation, Chinese vs. non-Chinese population: ROR 0.23, 95% CI 0.08 to 0.65; low risk in blinding of participants and personnel ROR 0.46, 95% CI 0.26 to 0.82; low risk in blinding of outcome assessment, ROR 0.50, 95% CI 0.28 to 0.87; and low risk in incomplete outcome data, ROR 0.54, 95% CI 0.32 to 0.93, Table 4).

**Table 4:** Discrepancies of treatment effects of Chinese versus non-Chinese population# in studies with a low risk of bias in each domain

ROB domain with low risk of bias	No. reviews	No. RCTs (Chinese^#^ vs. non-Chinese population)	No. sample size (Chinese^#^ vs. non-Chinese population)	ROR^*^ and 95% CI
Random sequence generation	4	13 vs. 8	1885 vs. 529	**0.23 [0.08, 0.65]** ^*^
Allocation concealment	2	3 vs. 4	1002 vs. 257	0.54 [0.15, 1.87]
Blinding of participants and personnel	2	4 vs. 22	410 vs. 2765	**0.46 [0.26, 0.82]** ^*^
Blinding of outcome assessment	3	5 vs. 21	1193 vs. 2723	**0.50 [0.28, 0.87]** ^*^
Incomplete outcome data	5	9 vs. 16	1505 vs. 1090	**0.54 [0.32, 0.93]** ^*^
Selective reporting	1	3 vs. 4	279 vs. 315	0.44 [0.15, 1.26]

^#^ Studies addressing Chinese populations published in both Chinese or other languages were included. ^*^ ROR less than 1 implies that acupuncture is more effective in Chinese populations than in non-Chinese populations

## Discussion

### Principal findings

Our findings suggest that an acupuncture related systematic review can increase the number of eligible studies and provide more information on the outcomes by extending the search to Chinese databases. Although the higher risk of bias could lead to an overestimation of treatment effects of acupuncture, we found no evidence that the higher risk of bias would increase the effect size of acupuncture in Chinese-language studies more often than in non-Chinese-language studies. We further tested the difference of treatment effects of acupuncture between different population groups and found acupuncture appearing to be more effective in Chinese populations than in other populations.

### Strengths and limitations

Strengths of our study include rigour of data abstraction and analysis, and the exploration of the influence of different types of risk of bias on the effect size of outcomes between Chinese- and non-Chinese-language acupuncture studies. We also compared the effect sizes between Chinese and non-Chinese populations. The study provides evidence for confirming the benefits and harms of including Chinese-language studies in acupuncture SR/MAs. We analyzed SR/MAs from Cochrane Library which is the database of largest collection of high-quality SR/MAs worldwide. The findings from this study are likely to be generalizable to acupuncture SR/MAs published elsewhere as well.

One limitation of this study is that we did not conduct our own detailed assessment of risk of bias to verify the authors’ ratings. Another limitation is that, in some Cochrane acupuncture reviews, the small number of studies in one of the language groups led to a very wide confidence interval of the ROR and made it challenging to interpret the results. To minimize this bias, we separately pooled the overall RORs for primary and secondary outcomes. Finally, we found a high risk of bias in Chinese-language studies than in studies published in other languages, however, we could not determine whether this risk was different between studies only indexed in Chinese versus international databases.

### Comparison with other studies

One prior study investigated 32 Cochrane reviews on acupuncture and suggested that searching Chinese databases did not increase the odds of positive conclusions in meta analyses (OR 0.51, 95% CI 0.11 to 2.44) when compared with not searching, and the odds of positive conclusions were also similar in Chinese-language acupuncture trials than in trials published in other languages (OR 1.29, 95% CI 0.26 to 6.49) [71]. Another previous study found that 60% of Cochrane acupuncture reviews did not search any Chinese database and inconclusive findings were more common in reviews that did not search any Chinese database [72]. In contrast to our study, none of the prior studies addressed the impact of risk of bias and population difference to the treatment effect of acupuncture.

### Implications

Our findings suggest that investigators should balance the benefits and harms when deciding about the inclusion of Chinese-language studies indexed in Chinese databases for an acupuncture SR/MA. Although searching Chinese databases can substantially increase the number of eligible studies and sample size in acupuncture reviews, the potentially higher risk of bias is an argument that needs to be considered in the inclusion of Chinese-language studies. Patients, investigators, and guideline panels should be cautious when adopting evidence from acupuncture reviews where studies with a high risk of bias contributed with a high weight to the meta-analysis.

We also observed larger treatment effects of acupuncture in Chinese-language studies than in studies published in other languages. Although the treatment effects of acupuncture tended to be greater in studies with a high risk of bias, this potential overestimation did not differ between studies published in Chinese and in other languages. In other words, the larger treatment effects in Chinese-language studies cannot be explained by a high risk of bias. Furthermore, our study found acupuncture to be more effective in Chinese populations than in other populations, which could at least partly explain the larger treatment effects observed in Chinese-language studies.

## Conclusion

Including Chinese-language trials in SR/MAs on acupuncture can increase the statistical power and provide more outcome data. A high risk of bias may lead to an overestimation of the treatment effects of acupuncture, but the degree of overestimation did not differ between Chinese-language studies and studies published in other languages. The difference in treatment effects of acupuncture was probably rather associated with differences in the study populations. Our findings can thus help understand the relationship between the risk of bias, language of publication, study population, and treatment effects in acupuncture related systematic reviews.

## Electronic supplementary material

Below is the link to the electronic supplementary material.

Supplementary Material 1

## Data Availability

The datasets analysed during this study are available from the corresponding author on reasonable request.

## Abbreviations

SRs: Systematic reviews
MAs: Meta-analyses
ROR: Ratio of odds ratio
CI: Confidence interval
OSF: Open Science Framework
ROB: Risk of bias
