# Supplementary material for: The relationship of publication language, study population, risk of bias, and treatment effects in acupuncture related systematic reviews: a meta-epidemiologic study
Source: BMC Med Res Methodol. 2023 Apr 20;23:96. doi: 10.1186/s12874-023-01904-w (PMC10120256; doi:10.1186/s12874-023-01904-w)
Supplement: Supplementary file 1 — Supplementary Material 1 [file 12874_2023_1904_MOESM1_ESM.docx]

**Supplementary Appendices**

**The relationship of publication language, study population, risk of bias, and treatment effects in acupuncture related systematic reviews: A meta-epidemiologic Study**

Contents

Appendix 1. Search strategy (December 1, 2021)3

Appendix Figure 1. The process of selecting study4

Appendix Figure 2. The characteristics of included studies4

Appendix Table 1. The characteristics of included acupuncture Cochrane reviews5

Appendix Figure 3. The information of Chinese language database14

Appendix Table 2. The selected outcomes for quantitative analysis15

Appendix Table 3. 21 reviews only included Chinese-language trials for binary outcome15

Appendix Figure 4. Meta-analysis estimates between Chinese- and non-Chinese-language acupuncture studies in each Cochrane review (Primary outcome)16

Appendix Figure 5. Meta-analysis estimates between Chinese- and non- Chinese-language acupuncture studies in each Cochrane review (Secondary outcome)16

Appendix Table 4. Risk of bias in Chinese-language RCTs and non-Chinese-language RCTs17

Appendix Table 5. The ROB items of Chinese-language acupuncture studies (n=319)18

Appendix Table 6. The ROB items of non-Chinese-language acupuncture studies (n=352)23

**Appendix 1. Search strategy (December 1, 2021)**

#1 MeSH descriptor: [Acupuncture] explode all trees

#2 MeSH descriptor: [Acupuncture Analgesia] explode all trees

#3 MeSH descriptor: [Acupuncture Points] explode all trees

#4 MeSH descriptor: [Acupuncture Therapy] explode all trees

#5 MeSH descriptor: [Acupuncture, Ear] explode all trees

#6 MeSH descriptor: [Moxibustion] explode all trees

#7 MeSH descriptor: [Dry Needling] explode all trees

#8 MeSH descriptor: [Meridians] explode all trees

#9 #1 OR #2 OR #3 OR #4 OR #5 OR #6 OR #7 OR #8

#10 (acupunctur*):ti,ab,kw OR (acupressur*):ti,ab,kw OR (electroacupunctur*):ti,ab,kw OR (Electro-acupuncture):ti,ab,kw OR (Acupoint):ti,ab,kw

#11 (needling*):ti,ab,kw OR (Acupuncture Treatment):ti,ab,kw OR (Pharmacoacupuncture Treatment):ti,ab,kw OR (dry needling):ti,ab,kw OR (auricular acupuncture):ti,ab,kw

#12 (fire needl*):ti,ab,kw OR (warming needl*):ti,ab,kw OR (filiform needl*):ti,ab,kw

#13 #10 OR #11 OR #12

#14 #9 OR #13

149 Articles identified from the Cochrane Library

0 Excluded articles according to title and abstract

149 Remaining articles reviewed by full text

65 Excluded articles according to selection criteria

30 Not acupuncture

6 Withdraw

20 qualitative analysis

1 Overview of systematic reviews

8 No study was included

84 Meta-analysis included in this study

45 MAs involving Chinese-language studies

**
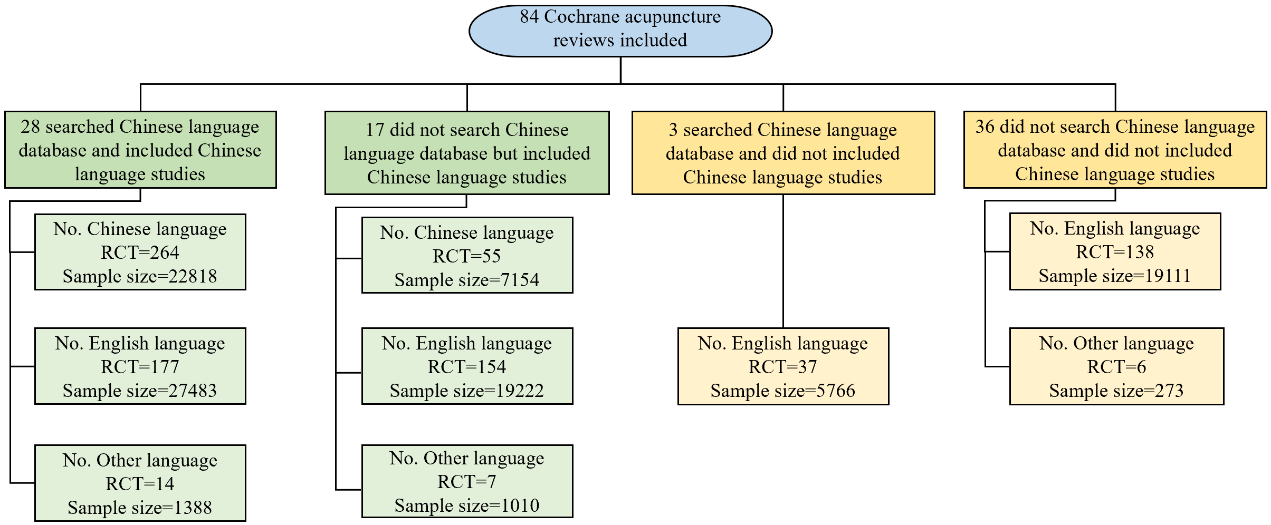
Appendix Figure 1. The process of selecting study.**

**Appendix Figure 2. The characteristics of included studies.**

**Appendix Table 1. The characteristics of included acupuncture Cochrane reviews.**

| **Number** | **Title** | **First author** | **Cochrane Group** | **Country (all authors)** | **Searching Chinese language database** | **Proportion of local language RCT** | **Included binary outcome analysis** |
| --- | --- | --- | --- | --- | --- | --- | --- |
| **Reviews that including Chinese-language studies (No.reviews=45)** | | | | | | | |
| #4 | Acupuncture and acupressure for premenstrual syndrome(1) | Armour 2018 | Cochrane Gynaecology and Fertility Group | Australia | Yes | 0.40 | Yes |
| #6 | Swallowing therapy for dysphagia in acute and subacute stroke(2) | Bath 2018 | Cochrane Stroke Group | UK | No | 0.45 | Yes |
| #13 | Complementary therapies for acne vulgaris(3) | Cao 2015 | Cochrane Skin Group | China, Australia, UK | Yes | 0.90 | No |
| #17 | Acupuncture for acute hordeolum(4) | Cheng 2017 | Cochrane Eyes and Vision Group | China, USA | Yes | 1.00 | No |
| #19 | Acupuncture for epilepsy(5) | Cheuk 2014 | Cochrane Epilepsy Group | China | Yes | 0.71 | Yes |
| #20 | Acupuncture for autism spectrum disorders (ASD)(6) | Cheuk 2011 | Cochrane Developmental, Psychosocial and Learning Problems Group | China | Yes | 0.40 | No |
| #22 | Acupuncture and related interventions for the treatment of symptoms associated with carpal tunnel syndrome(7) | Choi 2018 | Cochrane Neuromuscular Group | South Korea, USA | Yes | 0.42 | No |
| #24 | Cephalic version by moxibustion for breech presentation(8) | Coyle 2012 | Cochrane Pregnancy and Childbirth Group | Australia | No | 0.38 | No |
| #25 | Interventions for preventing mastitis after childbirth(9) | Crepinsek 2020 | Cochrane Pregnancy and Childbirth Group | Australia, UK | No | 1.00 | No |
| #26 | Acupuncture for restless legs syndrome(10) | Cui 2008 | Cochrane Movement Disorders Group | China | Yes | 1.00 | No |
| #28 | Acupuncture for treating fibromyalgia(11) | Deare 2013 | Cochrane Musculoskeletal Group | Australia, China | Yes | 0.11 | No |
| #39 | Non-pharmacological interventions for treating chronic  prostatitis/chronic pelvic pain syndrome(12) | Franco 2018 | Cochrane Urology Group | Argentina, Syrian Arab Republic, South Korea, China | No | 0.17 | No |
| #45 | Acupuncture for lateral elbow pain(13) | Green 2002 | Cochrane Musculoskeletal Group | Australia, Netherlands | No | 0.25 | No |
| #144 | Interventions for preventing nausea and vomiting in women undergoing regional anaesthesia for caesarean section(14) | Griffiths 2021 | Cochrane Pregnancy and Childbirth Group | Australia, UK | No | 0.07 | Yes |
| #58 | Complementary and miscellaneous interventions for nocturnal enuresis in children(15) | Huang 2011 | Cochrane Incontinence Group | China | Yes | 0.75 | Yes |
| #63 | Acupuncture for neuropathic pain in adults(16) | Ju 2017 | Cochrane Pain, Palliative and Supportive Care Group | China, UK | Yes | 0.50 | Yes |
| #65 | Acupuncture for symptomatic gastroparesis(17) | Kim 2018 | Cochrane Neuromuscular Group | South Korea | Yes | 0.97 | Yes |
| #66 | Acupuncture and related interventions for symptoms of chronic kidney disease(18) | Kim 2016 | Cochrane Kidney and Transplant Group | South Korea | Yes | 0.46 | Yes |
| #67 | Acupuncture for treating acute ankle sprains in adults(19) | Kim 2014 | Cochrane Bone, Joint and Muscle Trauma Group | South Korea, UK | Yes | 0.90 | No |
| #68 | Acupuncture for chronic hepatitis B(20) | Kong 2019 | Cochrane Hepato-Biliary Group | China, Denmark | Yes | 1.00 | No |
| #71 | Pain relief for women undergoing oocyte retrieval for assisted reproduction(21) | Kwan 2018 | Cochrane Gynaecology and Fertility Group | UK, Australia | No | 0.29 | No |
| #72 | Acupuncture for functional dyspepsia(22) | Lan 2014 | Cochrane Upper GI and Pancreatic Diseases Group | China | Yes | 0.71 | No |
| #74 | Stimulation of the wrist acupuncture point PC6 for preventing postoperative nausea and vomiting(23) | Lee 2015 | Cochrane Anaesthesia, Critical and Emergency Care Group | China | No | 0.02 | Yes |
| #79 | Acupuncture for polycystic ovarian syndrome(24) | Lim 2019 | Cochrane Gynaecology and Fertility Group | Australia | Yes | 0.38 | No |
| #83 | Acupuncture for treatment of irritable bowel syndrome(25) | Manheimer 2012 | Cochrane Inflammatory Bowel Disease and Functional Bowel Disorders Group | USA, China, Brazil | Yes | 0.65 | No |
| #84 | Acupuncture for hip osteoarthritis(26) | Manheimer 2018 | Cochrane Musculoskeletal Group | USA, China | No | 0.17 | No |
| #89 | Interventions for dysarthria due to stroke and other adultacquired, non-progressive brain injury(27) | Mitchell 2017 | Cochrane Stroke Group | UK | No | 1.00 | No |
| #93 | Interventions for improving sleep quality in people with chronic kidney disease(28) | Natale 2019 | Cochrane Kidney and Transplant Group | Sweden, Italy, Australia, New Zealand | No | 0.25 | No |
| #107 | Acupuncture for schizophrenia(29) | Shen 2014 | Cochrane Schizophrenia Group | China, UK | No | 0.80 | Yes |
| #146 | Interventions for the management of abdominal pain in Crohn’s disease and inflammatory bowel disease(30) | Sinopoulou 2021 | Cochrane Gut Group | UK, Qatar | No | 1.00 | No |
| #108 | Acupuncture or acupressure for induction of labour(31) | Smith 2017 | Cochrane Pregnancy and Childbirth Group | Australia | No | 0.05 | No |
| #109 | Acupuncture for depression(32) | Smith 2018 | Cochrane Common Mental Disorders Group | Australia, South Korea, China | Yes | 0.50 | Yes |
| #110 | Acupuncture for dysmenorrhoea(33) | Smith 2016 | Cochrane Gynaecology and Fertility Group | Australia, China | Yes | 0.48 | Yes |
| #113 | Acupuncture or acupressure for pain management during labour(34) | Smith 2020 | Cochrane Pregnancy and Childbirth Group | Australia, Singapore, Iran | No | 0.04 | No |
| #118 | Physical therapy for Bell’s palsy (idiopathic facial paralysis)(35) | Teixeira 2011 | Cochrane Neuromuscular Group | Brazil | No | 1.00 | No |
| #119 | Interventions for treating urinary incontinence after stroke in adults(36) | Thomas 2019 | Cochrane Incontinence Group | UK, Singapore | No | 0.80 | Yes |
| #120 | Acupuncture for neck disorders(37) | Trinh 2016 | Cochrane Back and Neck Group | Canada, Germany, Australia | Yes | 0.04 | No |
| #127 | Acupuncture for stress urinary incontinence in adults(38) | Wang 2013 | Cochrane Incontinence Group | China | Yes | 1.00 | No |
| #130 | Acupuncture and related interventions for smoking cessation(39) | White 2014 | Cochrane Tobacco Addiction Group | UK, Canada, China | Yes | 0.10 | Yes |
| #132 | Acupuncture for acute management and rehabilitation of traumatic brain injury(40) | Wong 2013 | Cochrane Injuries Group | China | Yes | 1.00 | No |
| #133 | Acupuncture for dysphagia in acute stroke(41) | Xie 2008 | Cochrane Stroke Group | China, USA | Yes | 1.00 | No |
| #134 | Acupuncture for acute stroke(42) | Xu 2018 | Cochrane Stroke Group | China | Yes | 0.58 | Yes |
| #135 | Acupuncture for stroke rehabilitation(43) | Yang 2016 | Cochrane Stroke Group | China | Yes | 0.94 | No |
| #136 | Acupuncture for hypertension(44) | Yang 2018 | Cochrane Hypertension Group | China, Canada | Yes | 0.82 | No |
| #142 | Chinese herbal medicine for primary dysmenorrhoea(45) | Zhu 2008 | Cochrane Gynaecology and Fertility Group | Australia, New Zealand | Yes | 1.00 | No |
| **Reviews that did not include Chinese-language studies (No.reviews=39)** | | | | | | | |
| #1 | Interventions for acne scars(46) | Abdel Hay 2016 | Cochrane Skin Group | Egypt, China, UK | No | NA | NA |
| #8 | Interventions for treating hyperemesis gravidarum(47) | Boelig 2016 | Cochrane Pregnancy and Childbirth Group | USA, UK, Italy | No | NA | NA |
| #14 | Acupuncture and electroacupuncture for the treatment of rheumatoid arthritis(48) | Casimiro 2005 | Cochrane Musculoskeletal Group | Canada, Australia | No | NA | NA |
| #18 | Acupuncture and assisted reproductive technology(49) | Cheong 2013 | Cochrane Menstrual Disorders and Subfertility Group | UK, China, New Zealand | Yes | NA | NA |
| #27 | Injected corticosteroids for treating plantar heel pain in adults(50) | David 2017 | Cochrane Bone, Joint and Muscle Trauma Group. | India, UK | No | NA | NA |
| #29 | Interventions (other than pharmacological, psychosocial or psychological) for treating antenatal depression(51) | Dennis 2013 | Cochrane Pregnancy and Childbirth Group | Canada, UK | No | NA | NA |
| #30 | Acupuncture for menopausal hot flushes(52) | Dodin 2013 | Cochrane Menstrual Disorders and Subfertility Group | Canada, UK, China | Yes | NA | NA |
| #32 | Management of gag reflex for patients undergoing dental treatment(53) | Eachempati 2019 | Cochrane Oral Health Group | Malaysia | No | NA | NA |
| #35 | Pharmacological treatment for antipsychotic-related constipation(54) | Every‐Palmer 2017 | Cochrane Schizophrenia Group | New Zealand, UK | No | NA | NA |
| #37 | Interventions for fatigue in inflammatory bowel disease(55) | Farrell 2020 | Cochrane IBD Group | Ireland, UK, Norway | No | NA | NA |
| #40 | Massage for low-back pain(56) | Furlan 2015 | Cochrane Back and Neck Group | Colombia, Canada, Brazil | No | NA | NA |
| #48 | Interventions for preventing nausea and vomiting in women undergoing regional anaesthesia for caesarean section(57) | Griffiths 2012 | Cochrane Pregnancy and Childbirth Group | Australia, UK | No | NA | NA |
| #50 | Exercises for mechanical neck disorders(58) | Gross 2015 | Cochrane Back Group | Canada, Netherlands, USA | No | NA | NA |
| #51 | Manipulation and mobilisation for neck pain contrasted against an inactive control or another active treatment(59) | Gross 2015 | Cochrane Back and Neck Group | Canada, USA, Netherlands | No | NA | NA |
| #145 | Autologous blood and platelet-rich plasma injection therapy for lateral elbow pain(60) | Karjalainen 2021 | Cochrane Musculoskeletal Group | Australia, Finland | No | NA | NA |
| #70 | Interventions for managing taste disturbances(61) | Kumbargere Nagraj 2017 | Cochrane Oral Health Group | Malaysia, USA, Nepal | No | NA | NA |
| #78 | Interventions for preventing and treating low-back and pelvic pain during pregnancy(62) | Liddle 2015 | Cochrane Pregnancy and Childbirth Group | Ireland, UK | No | NA | NA |
| #80 | Acupuncture for the prevention of tension-type headache(63) | Linde 2016 | Cochrane Pain, Palliative and Supportive Care Group | Germany, Italy, China, South Korea, USA, UK | No | NA | NA |
| #81 | Acupuncture for the prevention of episodic migraine(64) | Linde 2016 | Cochrane Pain, Palliative and Supportive Care Group | Germany, Italy, China, USA, UK | No | NA | NA |
| #82 | Acupuncture for peripheral joint osteoarthritis(65) | Manheimer 2010 | Cochrane Musculoskeletal Group | USA, China, Germany, South Korea, UK, Netherlands | No | NA | NA |
| #85 | Non-pharmacological interventions for assisting the induction of anaesthesia in children(66) | Manyande 2015 | Cochrane Anaesthesia Group | UK, Australia, New Zealand | No | NA | NA |
| #86 | Interventions for nausea and vomiting in early pregnancy(67) | Matthews 2015 | Cochrane Pregnancy and Childbirth Group | Ireland, USA, UK | No | NA | NA |
| #87 | Acupuncture for chronic asthma(68) | McCarney 2003 | Cochrane Airways Group | UK, Germany | No | NA | NA |
| #88 | Planned early birth versus expectant management (waiting) for prelabour rupture of membranes at term (37 weeks or more)(69) | Middleton 2017 | Cochrane Pregnancy and Childbirth Group | Australia, New Zealand | No | NA | NA |
| #90 | Platelet-rich therapies for musculoskeletal soft tissue injuries(70) | Moraes 2014 | Cochrane Bone, Joint and Muscle Trauma Group | Brazil | No | NA | NA |
| #92 | Psychosocial interventions for preventing and treating depression in dialysis patients(71) | Natale 2019 | Cochrane Kidney and Transplant Group | Italy, Australia, New Zealand | No | NA | NA |
| #94 | Non-surgical treatment (other than steroid injection) for carpal tunnel syndrome(72) | O'Connor 2003 | Cochrane Neuromuscular Disease Group | Australia, Canada | No | NA | NA |
| #100 | Massage for mechanical neck disorders(73) | Patel 2012 | Cochrane Back Group | Canada, USA | No | NA | NA |
| #102 | Interventions for heartburn in pregnancy(74) | Phupong 2015 | Cochrane Pregnancy and Childbirth Group | Thailand | No | NA | NA |
| #105 | Relaxation for perimenopausal and postmenopausal symptoms(75) | Saensak 2014 | Cochrane Menstrual Disorders and Subfertility Group | Thailand | No | NA | NA |
| #112 | Complementary and alternative therapies for pain management in labour(76) | Smith 2006 | Cochrane Pregnancy and Childbirth Group | Australia | No | NA | NA |
| #116 | Sucrose for analgesia in newborn infants undergoing painful procedures(77) | Stevens 2016 | Cochrane Neonatal Group | Canada | No | NA | NA |
| #117 | Shock wave therapy for rotator cuff disease with or without calcification(78) | Surace 2020 | Cochrane Musculoskeletal Group | Australia | No | NA | NA |
| #122 | Non-steroidal anti-inflammatory drugs for acute gout(79) | van Durme 2014 | Cochrane Musculoskeletal Group | Belgium, Netherlands, Australia, USA | No | NA | No |
| #123 | Conservative treatments for whiplash(80) | Verhagen 2007 | Cochrane Back and Neck Group | Netherlands | No | NA | NA |
| #125 | Pharmacological and mechanical interventions for labour induction in outpatient settings(81) | Vogel 2017 | Cochrane Pregnancy and Childbirth Group | Switzerland, Kenya, UK, Italy | No | NA | NA |
| #137 | Treatments for breast engorgement during lactation(82) | Zakarija-Grkovic 2020 | Cochrane Pregnancy and Childbirth Group | Croatia, UK | No | NA | NA |
| #140 | Acupuncture for pain in endometriosis(83) | Zhu 2011 | Cochrane Gynaecology and Fertility Group | Australia, USA | Yes | NA | NA |
| #143 | Complementary and alternative therapies for post-caesarean pain(84) | Zimpel 2020 | Cochrane Pregnancy and Childbirth Group | Brazil | No | NA | NA |

**Appendix Figure 3. The information of Chinese language database.**


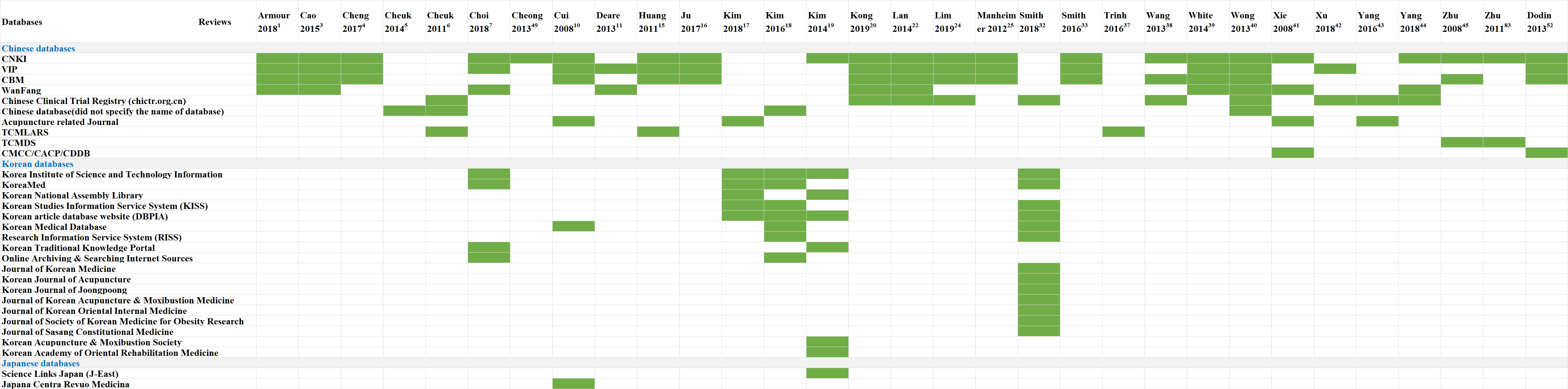


Notes: CNKI, China National Knowledge Infrastructure; CBM, Chinese Biomedical Literature database; TCMLARS, Traditional Chinese Medical Literature Analysis and Retrieval System; TCMDS, Traditional Chinese Medicine Database System; CMCC, Chinese Medical Current Content; CACP, Chinese Academic Conference Papers database; CDDB, Chinese Dissertations database.

**Appendix Table 2. The selected outcomes for quantitative analysis.**

| **Reviews** | **Outcomes** |
| --- | --- |
| **Primary outcome (n=9)** | |
| Xu 2018(42) | Death or dependency at end of follow-up |
| Smith 2016(33) | Pain relief |
| Huang 2011(15) | Failure or relapse of nocturnal enuresis |
| Lee 2015(23) | Vomiting |
| White 2014(39) | Short-term smoking cessation |
| Armour 2018(1) | Adverse events |
| Griffiths 2021(14) | Vomiting |
| Thomas 2019(36) | Number of participants continent after treatment |
| Cheuk 2014(5) | 50% or greater reduction in seizure frequency |
| **Secondary outcome (n=6)** | |
| Shen 2014(29) | Global state: Not improved |
| Ju 2017(16) | Any pain-related outcomes |
| Bath 2018(2) | Proportion of participants with dysphagia at end of trial |
| Smith 2018(32) | Remission of depression |
| Kim 2018(17) | Proportion of people whose symptoms 'improved' (4 to 12 weeks) |
| Kim 2016(18) | Uraemic pruritus improvement |

**Appendix Table 3. 21 reviews only included Chinese-language trials for binary outcome.**

| **Reviews** | **Outcomes** |
| --- | --- |
| **Primary outcome (n=15)** | |
| Cheng 2017(4) | Relief of hordeolum at short term |
| Cheuk 2011(6) | Frequency of improvement in Autism Behavior Checklist |
| Coyle 2012(8) | Non-cephalic presentation at birth |
| Crepinsek 2020(9) | Incidence of mastitis within 6 months postpartum |
| Cui 2008(10) | Symptom remission of restless legs syndrome |
| Deare 2013(11) | Adverse events |
| Green 2002(13) | Still with symptoms after treatment |
| Kim 2014(19) | Cure rate of acute ankle sprains |
| Manheimer 2012(25) | Symptom severity of irritable bowel syndrome(responders) |
| Sinopoulou 2021(30) | Withdrawals due to adverse events |
| Smith 2020(34) | Assisted vaginal birth |
| Teixeira 2011(35) | Number of participants without recovery |
| Wong 2013(40) | Frequency of post-treatment muscle strength grade IV toVI |
| Xie 2008(41) | Recovery of dysphagia in acute stroke |
| Zhu 2008(45) | Reduction of pain |
| **Secondary outcome (n=6)** | |
| Cao 2015(3) | Number of participants with remission (short-term data) |
| Choi 2018(7) | Responder rate |
| Kong 2019(20) | Proportion of participants with 1 or more adverse events considered not to be serious |
| Lim 2019(24) | Adverse events |
| Wang 2013(38) | Number of women's stress urinary incontinence improved (subjective) |
| Yang 2016(43) | Improvement of global neurological deficit at the end of treatment |


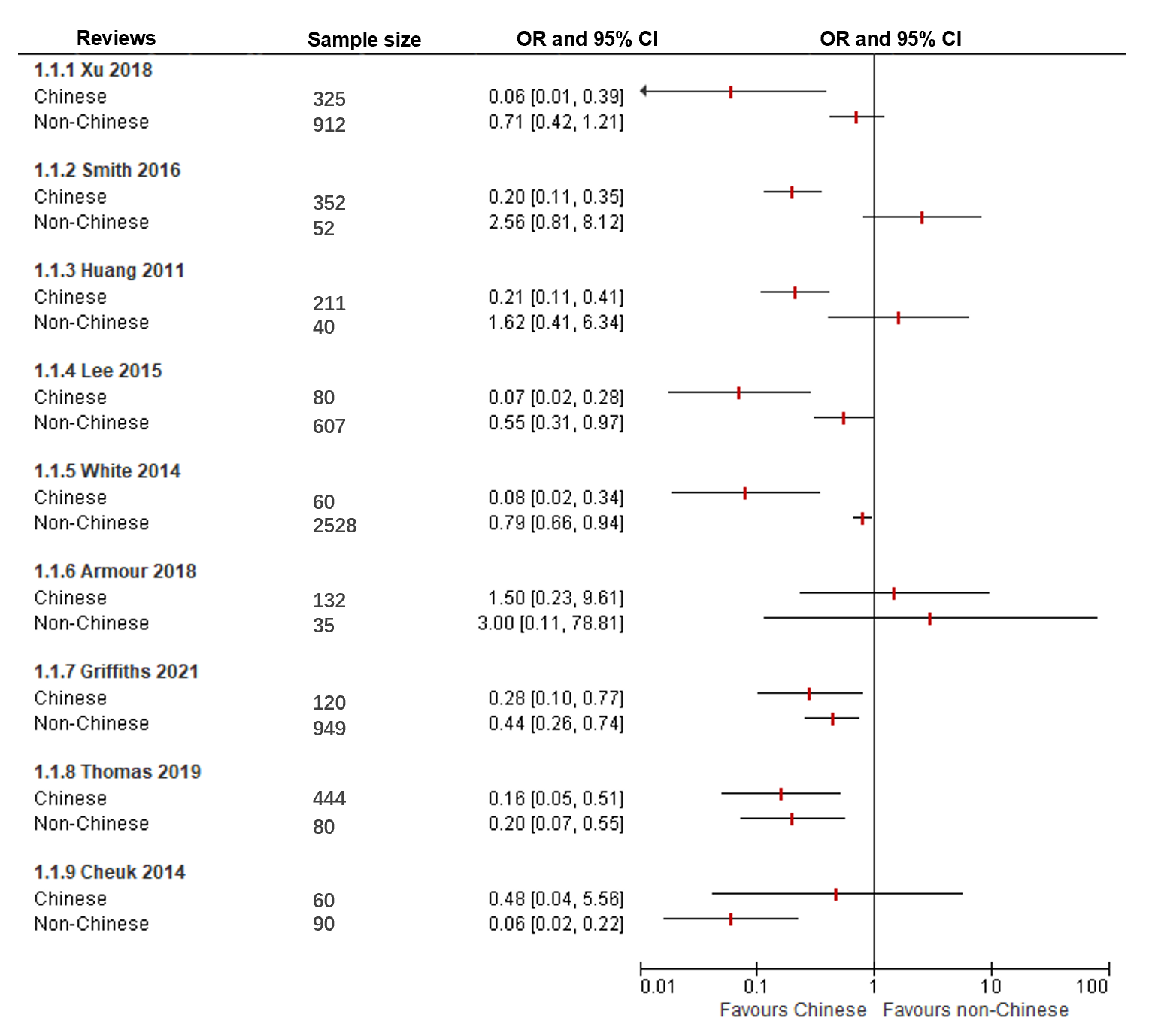
**Appendix Figure 4. Meta-analysis estimates between Chinese- and non-Chinese-language acupuncture studies in each Cochrane review (Primary outcome).**


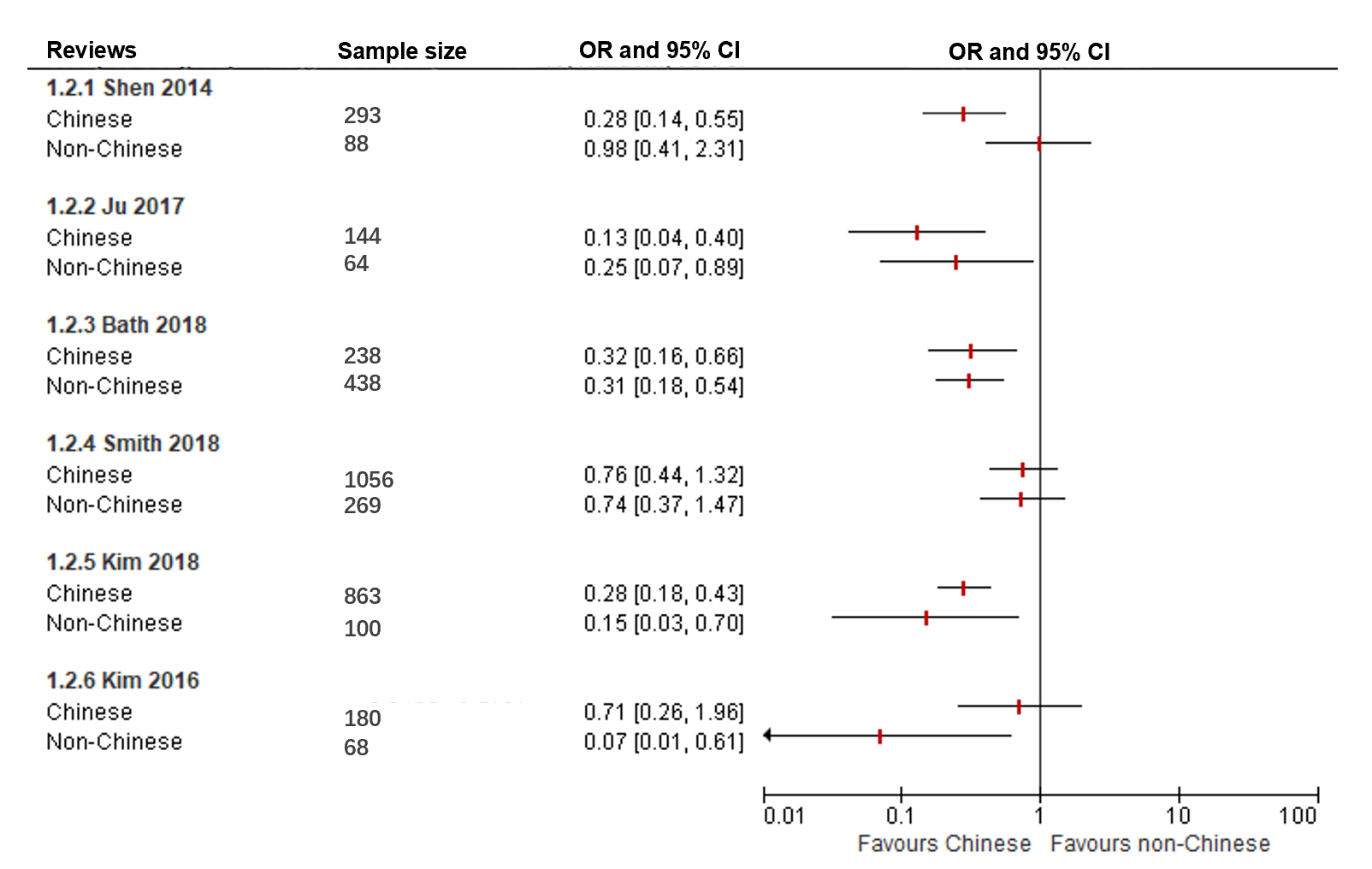


**Appendix Figure 5. Meta-analysis estimates between Chinese- and non-Chinese-language acupuncture studies in each Cochrane review (Secondary outcome).**

**Appendix Table 4. Risk of bias in Chinese-language RCTs and non-Chinese-language RCTs.**

| ROB items | Risk of bias | Chinese (No. RCT=319) | English (No. RCT=352) |
| --- | --- | --- | --- |
| Random sequence generation (selection bias), n (%) | high risk | 23(7) | 12(3) |
|  | low risk | 171(54) | 214(61) |
|  | unclear | 125(39) | 126(36) |
| Allocation concealment (selection bias), n (%) | high risk | 26(8) | 15(4) |
|  | low risk | 32(10) | 124(35) |
|  | unclear | 261(82) | 213(61) |
| Blinding of participants and personnel (performance bias), n (%) | high risk | 218(68) | 116(33) |
|  | low risk | 10(3) | 173(49) |
|  | unclear | 91(29) | 63(18) |
| Blinding of outcome assessment (detection bias), n (%) | high risk | 115(36) | 64(18) |
|  | low risk | 22(7) | 187(53) |
|  | unclear | 182(57) | 101(29) |
| Incomplete outcome data (attrition bias), n (%) | high risk | 21(7) | 56(16) |
|  | low risk | 208(65) | 236(67) |
|  | unclear | 90(28) | 60(17) |
| Selective reporting (reporting bias), n (%) | high risk | 32(10) | 33(9) |
|  | low risk | 75(24) | 102(29) |
|  | unclear | 212(66) | 217(62) |

**Appendix Table 5. The ROB items of Chinese-language acupuncture studies (n=319).**

| Included study | Random sequence generation (selection bias) | Allocation concealment (selection bias) | Blinding of participants and personnel (performance bias) | Blinding of outcome assessment (detection bias) | Incomplete outcome data (attrition bias) | Selective reporting (reporting bias) |
| --- | --- | --- | --- | --- | --- | --- |
| Yu 2006 | + | + | + | + | + | ? |
| Zhang 2017 | + | + | + | + | + | + |
| Sun 2005 | + | ? | - | ? | + | ? |
| Wang 1997 | ? | ? | ? | ? | + | ? |
| Pan 2002 | ? | ? | - | + | - | ? |
| Wang 2000 | + | ? | - | + | + | ? |
| Yang 2000 | ? | ? | - | ? | + | ? |
| Xue 1987 | ? | ? | - | ? | + | ? |
| Chen 2006 | ? | ? | - | ? | + | ? |
| Chen 2008 | + | ? | - | ? | - | ? |
| Cui 2000 | + | ? | - | ? | + | ? |
| Ding 2005 | ? | ? | - | ? | + | ? |
| Wang 2005 | ? | ? | - | ? | + | ? |
| Xiong 2010 | ? | ? | - | + | + | ? |
| Yao 2006 | + | ? | - | ? | - | ? |
| Zhang 2001 | + | ? | - | ? | + | ? |
| Ma 1999 | ? | ? | ? | ? | + | ? |
| Zhang 1991 | ? | ? | - | + | + | ? |
| Liu 2010 | + | ? | - | ? | - | ? |
| Luo 2006 | ? | ? | ? | ? | + | ? |
| Ma 2008 | ? | ? | - | ? | + | ? |
| Tang 2005 | ? | ? | - | ? | + | ? |
| Wang 2006 | + | ? | - | ? | + | ? |
| Xu 2004 | + | ? | - | ? | + | ? |
| Zhao 2005a | ? | ? | - | ? | + | - |
| Zhao 2005b | ? | ? | - | ? | + | - |
| Cheng 2007 | ? | ? | - | ? | + | ? |
| Ding 2003 | + | ? | - | ? | + | ? |
| Dong 2007 | ? | ? | - | ? | ? | ? |
| Du 2005 | + | ? | - | - | - | ? |
| Duan 2008 | + | ? | - | ? | + | ? |
| Fu 2008 | + | + | ? | ? | - | ? |
| Han 2002 | ? | ? | - | ? | + | ? |
| He 2005 | + | ? | - | ? | + | ? |
| He 2007 | ? | ? | - | ? | + | ? |
| Huang 2013 | + | ? | - | - | + | ? |
| Li 2004 | + | ? | - | ? | ? | ? |
| Li 2007 | ? | ? | - | ? | + | - |
| Li 2008 | + | ? | + | ? | + | ? |
| Li 2011b | + | ? | + | + | + | ? |
| Lin 2012 | + | ? | - | - | + | ? |
| Liu 2006 | + | ? | - | - | ? | ? |
| Liu 2015 | + | ? | - | - | ? | ? |
| Luo 1988 | ? | ? | - | ? | ? | ? |
| Lv 2015 | + | ? | - | ? | ? | ? |
| Ma 2011 | + | ? | - | - | ? | ? |
| Pei 2006 | + | ? | - | - | ? | ? |
| Qiao 2007 | + | ? | - | - | + | ? |
| Sun 2010 | + | ? | - | - | + | ? |
| Sun 2015b | + | ? | - | - | ? | ? |
| Tang 2003 | + | ? | - | - | ? | ? |
| Wang 2015 | + | + | - | ? | ? | ? |
| Xiao 2014 | + | ? | - | - | ? | ? |
| Xu 2011 | + | + | - | - | ? | ? |
| Yan 2004 | ? | ? | - | ? | + | ? |
| Zhang 2005a | + | ? | - | - | + | ? |
| Zhang 2007a | + | - | - | - | ? | ? |
| Zhuang 2004 | + | ? | - | ? | + | ? |
| Cai 2002 | ? | ? | ? | ? | ? | ? |
| Chen 1997 | + | + | ? | ? | ? | ? |
| Chen 2007 | ? | ? | ? | ? | ? | ? |
| Chen 2015 | ? | ? | ? | ? | ? | ? |
| Dong 2006 | + | ? | ? | ? | ? | ? |
| Guo 2016 | + | ? | ? | ? | + | ? |
| Huang 2002 | ? | ? | ? | ? | ? | ? |
| Jin 1999 | ? | ? | ? | ? | ? | ? |
| Lin 2005 | ? | ? | ? | ? | ? | ? |
| Mu 2008 | ? | ? | ? | ? | ? | ? |
| Ou 2014 | ? | ? | ? | ? | ? | ? |
| Pang 2006 | ? | ? | ? | ? | ? | ? |
| Shen 2012a | + | ? | ? | ? | ? | ? |
| Wang 2008 | ? | ? | ? | ? | ? | ? |
| Wu 2002 | ? | ? | ? | + | + | ? |
| Yu 1993 | + | + | ? | ? | ? | ? |
| Zhang 2005a | + | + | ? | ? | ? | ? |
| Zhang 2013 | ? | ? | ? | ? | ? | ? |
| Zhu 2007 | ? | ? | ? | ? | ? | ? |
| Deng 2001a | + | ? | - | - | + | + |
| Leng 2000 | ? | ? | - | - | + | + |
| Li 2007 | + | + | - | - | + | + |
| Ma 2001 | ? | ? | - | - | + | + |
| Mao 2011 | + | ? | - | - | + | + |
| Shi 2001 | ? | ? | - | - | + | + |
| Yi 2009 | + | ? | - | - | + | + |
| Yu 1999 | ? | ? | - | - | + | + |
| Zhang 2006a | - | - | - | - | + | + |
| Zhang 2006b | + | + | - | - | + | + |
| Zhuang 2004 | ? | ? | - | - | + | + |
| Zhuang 2006 | - | - | - | - | + | + |
| Han 2017a | + | ? | - | - | + | ? |
| Wang 2016 | ? | ? | - | - | + | ? |
| Zhao 2016 | ? | ? | - | - | + | ? |
| Cheng 2003 | + | ? | - | ? | + | ? |
| Ge 2010 | + | ? | - | ? | + | ? |
| Ge 2015 | ? | ? | - | ? | + | ? |
| He 2006 | + | ? | - | ? | + | ? |
| Kong 2009 | + | ? | - | ? | + | ? |
| Li 2013 | + | ? | - | ? | + | ? |
| Lin 2013 | + | ? | - | ? | + | ? |
| Liu 2001 | ? | ? | - | ? | + | ? |
| Liu 2009 | ? | ? | - | ? | + | ? |
| Liu 2011 | ? | ? | - | ? | + | ? |
| Liu 2012 | + | + | - | ? | + | ? |
| Mo 2005 | ? | ? | - | ? | + | ? |
| Mu 2010 | ? | ? | - | ? | + | ? |
| Qi 2013 | ? | ? | - | ? | + | ? |
| Shi 2012 | + | + | ? | + | + | + |
| Wang 2003 | ? | ? | - | ? | + | ? |
| Wang 2007 | ? | ? | - | ? | + | ? |
| Wang 2009 | ? | ? | - | ? | + | ? |
| Wu 2015 | ? | ? | - | ? | + | ? |
| Xu 2012 | + | ? | - | ? | + | ? |
| Xu 2016 | ? | ? | - | ? | + | ? |
| Xue 2014 | + | ? | - | ? | + | ? |
| Yang 2013 | ? | ? | - | ? | + | ? |
| Yuan 2004 | ? | ? | - | ? | ? | ? |
| Zeng 2006 | + | ? | - | ? | + | ? |
| Zeng 2008 | + | ? | - | ? | + | ? |
| Zhang 2007 | + | ? | - | ? | + | ? |
| Zhang 2013 | + | + | - | ? | + | ? |
| Zhang 2014 | + | ? | - | ? | + | ? |
| Zhao 2004 | + | ? | - | ? | + | ? |
| Zhao 2011 | ? | ? | - | ? | + | ? |
| Huang 2012 | - | - | + | + | + | ? |
| Li 2009 | ? | ? | ? | ? | + | ? |
| Han 2006 | ? | ? | ? | ? | + | ? |
| Bai 2007i | - | ? | ? | - | ? | ? |
| Bai 2007ii | - | ? | ? | - | ? | ? |
| Han 2004 | + | + | ? | + | + | ? |
| Huang 2010 | ? | ? | ? | ? | ? | ? |
| Wei 2005 | ? | ? | ? | + | ? | ? |
| Chen 2004 | - | ? | + | + | + | ? |
| Lin 2002 | - | ? | + | + | + | ? |
| Yang 2006 | - | ? | + | + | + | ? |
| Chu 2011 | + | ? | ? | ? | + | ? |
| Liu 2006 | ? | ? | ? | ? | + | ? |
| Song 2013 | + | ? | ? | ? | ? | ? |
| Zhang 2002 | ? | ? | ? | ? | + | ? |
| Zhu 2010 | + | ? | - | ? | + | + |
| Chang 2010 | + | + | ? | + | - | + |
| Jin 2011 | + | + | + | + | + | - |
| Shi 2009 | + | ? | - | ? | + | - |
| Tang 2006 | + | ? | - | ? | - | + |
| Yang 2009 | + | ? | - | ? | - | + |
| Cao 2017 | + | ? | - | - | - | + |
| Du 2011 | + | + | - | - | + | - |
| Jin 2016 | + | ? | - | - | - | + |
| An 2010 | + | + | - | - | ? | + |
| Chen 2009 | - | - | - | - | ? | + |
| Chen 2011 | + | + | - | - | + | + |
| Li 2009 | - | - | - | - | ? | + |
| Liu 1997 | + | ? | - | - | - | + |
| Liu 2010 | ? | - | - | - | ? | + |
| Shi 2010 | + | + | - | - | + | + |
| Sun 2011 | + | - | - | - | + | + |
| Xiong 2008a | + | - | - | - | - | + |
| Xue 2009 | ? | ? | - | - | + | + |
| Zeng 2010 | + | + | - | - | + | + |
| Sheng 2010 | ? | ? | - | - | + | + |
| Cao 2011 | + | ? | ? | ? | + | ? |
| Han 2012 | + | + | ? | ? | + | ? |
| Hu 2005 | + | ? | - | - | ? | + |
| Li 2008 | + | ? | - | - | + | ? |
| Li 2012b | + | ? | - | - | + | ? |
| Peng 2012 | ? | ? | - | - | + | + |
| Qiao 2013 | + | ? | - | - | + | + |
| Ruan 2011 | + | + | - | - | + | + |
| Song 2013 | + | + | - | - | + | ? |
| Sun 2011 | + | ? | - | - | + | + |
| Wang 2005b | + | ? | - | - | + | ? |
| Wang 2013b | + | ? | - | - | + | + |
| Wang 2014a | + | ? | ? | ? | + | ? |
| Xu 2013 | + | ? | ? | ? | + | ? |
| Xu 2014 | + | ? | - | - | + | ? |
| Yu 2014 | + | ? | - | - | + | ? |
| Zhang 2012 | + | ? | ? | ? | + | ? |
| Zhang 2013a | + | ? | - | - | + | ? |
| Zhang 2013b | + | ? | ? | ? | + | ? |
| Zhi 2007 | + | ? | - | - | + | ? |
| Huang 2008 | + | ? | - | ? | + | ? |
| Liang 2009 | ? | ? | ? | ? | + | + |
| Chen 2000 | ? | ? | - | - | + | + |
| Chen 2006a | + | ? | - | - | + | + |
| Chen 2010a | + | ? | - | - | + | + |
| Dan 1998 | ? | ? | ? | ? | + | + |
| Guo 2009 | + | ? | - | - | + | + |
| Hao 2006 | ? | ? | ? | ? | + | ? |
| Li 2012a | ? | ? | - | - | + | + |
| Liao 2006 | + | ? | - | - | + | + |
| Liu 2011 | ? | ? | - | - | + | + |
| Ma 2011 | + | ? | - | - | + | + |
| Shen 2010a | + | ? | - | - | + | + |
| Tian 2007 | + | ? | - | - | + | + |
| Wang 2012a | + | ? | - | - | - | + |
| Wu 2003 | + | ? | - | - | + | + |
| Wu 2011 | + | ? | - | - | + | + |
| Xie 2004 | + | ? | - | - | + | + |
| Yang 2010a | + | ? | ? | ? | + | + |
| Zhang 2012a | + | ? | - | - | + | + |
| Bao 2012 | + | ? | ? | ? | ? | ? |
| Dai 1997 | ? | ? | ? | ? | ? | ? |
| Gao 2014a | + | ? | ? | ? | + | ? |
| Guo 2011 | ? | ? | ? | ? | ? | ? |
| Guo 2012 | ? | ? | ? | ? | ? | ? |
| Huang 2008a | + | ? | ? | ? | ? | ? |
| Ke 2015 | ? | ? | ? | ? | ? | ? |
| Li 1997a | ? | ? | ? | ? | ? | ? |
| Li 2010a | + | ? | ? | ? | + | ? |
| Li 2011a | ? | ? | ? | ? | ? | ? |
| Li 2013a | ? | ? | ? | ? | ? | ? |
| Liu 2013a | ? | ? | ? | ? | ? | ? |
| Lun 1999 | ? | ? | ? | ? | ? | ? |
| Sun 2013a | + | ? | ? | ? | + | ? |
| Sun 2015 | + | ? | ? | ? | ? | ? |
| Wang 2001 | ? | ? | ? | ? | ? | ? |
| Wang 2011a | + | ? | ? | ? | ? | ? |
| Wang 2012 | + | ? | ? | ? | ? | ? |
| Wu 2008 | ? | ? | ? | ? | ? | ? |
| Wu 2011a | + | ? | ? | ? | ? | ? |
| Wu 2013a | + | ? | ? | ? | ? | ? |
| Xu 2013 | ? | ? | ? | ? | ? | ? |
| Yao 2014 | + | ? | ? | ? | ? | ? |
| Zhan 2014 | + | ? | ? | ? | + | ? |
| Zhang 2013a | + | + | - | ? | ? | ? |
| Zhang 2015 | + | ? | ? | ? | ? | ? |
| Zheng 2014 | + | ? | ? | ? | + | ? |
| Zhou 2014 | + | + | ? | + | + | ? |
| Zhu 2007 | ? | ? | ? | ? | ? | ? |
| Guo 2005 | - | - | ? | ? | + | ? |
| Feng 2011 | ? | ? | - | ? | + | ? |
| Jin 2011 | ? | ? | - | ? | + | ? |
| Li 2011 | ? | ? | - | ? | + | ? |
| Ramin 2013 | ? | + | - | - | + | ? |
| Zhang 2011 | + | ? | + | + | + | + |
| Wang 2007 | - | - | - | - | + | + |
| Wong 2008 | + | ? | - | - | + | + |
| Yan 2007 | - | - | - | - | + | + |
| Zhou 2008 | ? | ? | - | - | + | + |
| Wang 1997 | + | ? | - | - | + | ? |
| Cheng 2012 | ? | ? | - | ? | - | ? |
| Cui 2012 | - | - | - | ? | ? | ? |
| Dai 2007a | ? | ? | - | ? | ? | ? |
| Rui 2002 | - | - | - | ? | ? | ? |
| Song 2007 | - | - | - | ? | ? | ? |
| Sun 2008a | + | ? | - | ? | - | ? |
| Sun 2012 | ? | ? | - | ? | ? | ? |
| Xie 2012 | ? | ? | - | ? | ? | ? |
| Zhang 2011d | ? | ? | - | ? | + | ? |
| Zhao 1995 | ? | ? | - | ? | - | ? |
| Zhao 2011 | ? | ? | - | ? | ? | ? |
| Huang 2006 | + | + | - | ? | + | ? |
| Han 2010 | + | ? | - | ? | - | ? |
| Wen 2012 | + | ? | - | ? | ? | ? |
| Liu 2006 | + | ? | - | ? | ? | ? |
| Liu 2007 | + | ? | - | ? | + | ? |
| Liu 2008 | + | ? | - | ? | ? | ? |
| You 2012 | + | ? | - | ? | ? | ? |
| Zhang 2010 | + | ? | - | ? | - | ? |
| Ni 2008 | + | ? | - | ? | + | ? |
| Zhao 2011 | ? | ? | - | ? | ? | ? |
| Dai 2007a | ? | ? | - | - | ? | + |
| Chen 2009 | + | ? | - | - | + | ? |
| Hui 2006 | + | ? | ? | ? | ? | ? |
| Jiang 2000 | + | ? | ? | ? | + | ? |
| Mao 1998 | + | ? | - | ? | + | ? |
| Chen 2012 | ? | ? | - | - | + | - |
| Ge 2000 | ? | ? | - | - | + | - |
| Hao 2006 | ? | ? | - | - | + | - |
| Jian 2004 | + | ? | - | - | + | - |
| Jiang 2011 | - | - | - | - | ? | - |
| Ni 2010 | ? | ? | - | - | + | - |
| Ruan 1995 | ? | ? | - | - | + | - |
| Shi 2013 | - | - | - | - | + | - |
| Sun 2011 | ? | ? | - | - | + | - |
| Wang 2009 | ? | ? | - | - | ? | - |
| Wei 2004 | - | - | - | - | ? | - |
| Wu 2006 | ? | ? | - | - | + | - |
| Wu 2007 | ? | ? | - | - | + | - |
| Yu 1996 | ? | ? | - | - | + | - |
| Yu 1999 | ? | ? | - | - | + | - |
| Zhang 2011 | - | - | - | - | + | - |
| Zhang 2012 | ? | ? | - | - | + | - |
| Zhou 2008 | ? | ? | - | - | + | - |
| Meng 2008 | + | ? | ? | ? | + | + |
| Meng 2009 | + | ? | ? | ? | ? | + |
| Long 1994 | ? | ? | ? | ? | ? | ? |
| Han 2004 | + | + | - | - | + | ? |
| Cao 2001 | ? | ? | - | - | - | + |
| Chang 2005 | ? | ? | - | - | + | + |
| Ding 2002 | ? | ? | - | - | + | + |
| Song 2007 | ? | ? | - | - | + | + |
| Shi 2003 | - | - | - | ? | ? | ? |
| Zhou 2002 | + | + | - | ? | ? | ? |
| Li 2006a | + | ? | ? | - | ? | ? |
| Pang 2009 | + | - | ? | + | - | ? |
| Qi 2013 | + | - | ? | + | + | ? |
| Xu 2004 | - | - | ? | ? | ? | ? |
| Yang 2014 | + | - | ? | - | + | ? |
| Zhang 1991 | ? | ? | ? | + | + | ? |
| Bi 2007 | + | - | - | - | + | + |
| He 2015 | ? | ? | ? | ? | + | ? |
| Miao 2001 | + | ? | ? | ? | ? | ? |
| Wang 2003 | + | ? | - | ? | ? | ? |
| Zhang 2005 | ? | ? | - | - | + | + |
| Qu 2005 | - | - | - | - | + | + |
| Wong 2004 | + | + | - | - | + | + |
| Pan 2004 | ? | ? | - | - | + | + |
| Yang 2001 | ? | ? | - | - | + | + |
| Xu 2010 | ? | ? | ? | ? | + | ? |
| Huang 2011 | + | ? | - | ? | + | - |
| Jing 2016 | + | ? | - | ? | + | - |
| Liao 2011 | + | + | - | ? | + | - |
| Wang 2002 | + | ? | - | ? | + | - |
| Wang 2013 | + | ? | - | ? | + | - |
| Zhang 2014 | + | ? | - | ? | + | - |
| Zhu 2016 | + | ? | - | ? | + | - |
| Zou 2011 | + | ? | - | ? | + | - |
| Li 2012 | ? | ? | ? | ? | + | ? |
| Bao 2016 | + | ? | ? | ? | + | + |

**Appendix Table 6. The ROB items of non-Chinese-language acupuncture studies (n=352).**

| Included study | Language | Random sequence generation (selection bias) | Allocation concealment (selection bias) | Blinding of participants and personnel (performance bias) | Blinding of outcome assessment (detection bias) | Incomplete outcome data (attrition bias) | Selective reporting (reporting bias) |
| --- | --- | --- | --- | --- | --- | --- | --- |
| Bazarganipour 2017 | English | + | ? | + | ? | + | ? |
| Habek 2002 | English | ? | ? | ? | ? | + | ? |
| Shin 2009 | English | ? | ? | - | ? | - | ? |
| Cheng 2009 | English | + | + | + | + | + | ? |
| Zhang 1987 | English | ? | ? | - | ? | + | ? |
| Zhang 1993 | English | ? | ? | - | ? | - | ? |
| Zhou 1997 | English | ? | ? | - | + | + | ? |
| Liu 1986 | English | ? | ? | - | ? | + | ? |
| Bouhlel 2011 | French | ? | ? | + | + | - | ? |
| Allen 1998 | English | + | + | + | ? | + | ? |
| Allen 2006 | English | ? | + | + | + | - | ? |
| Andreescu 2011 | English | + | + | + | + | + | ? |
| Bosch 2015 | English | + | ? | - | ? | - | ? |
| Chung 2015 | English | + | + | + | + | + | ? |
| Duan 2011 | English | + | ? | - | + | + | ? |
| Eich 2000 | German | ? | ? | + | ? | + | ? |
| Fan 2005 | English | + | + | - | ? | - | ? |
| Fan 2013 | English | + | ? | - | ? | + | ? |
| Feng 2011 | English | + | ? | - | ? | + | ? |
| Fu 2006 | English | + | + | + | ? | ? | ? |
| He 2012 | English | + | - | - | - | ? | ? |
| Liu 2013a | English | + | ? | - | - | + | ? |
| Luo 1985 | English | ? | ? | - | ? | + | ? |
| Luo 1998 | English | ? | ? | - | ? | + | ? |
| Ma 2012 | English | + | + | - | - | ? | ? |
| MacPherson 2013 | English | + | + | - | ? | + | + |
| Qu 2013 | English | + | + | - | + | + | ? |
| Quah-Smith 2005 | English | + | + | + | + | - | ? |
| Quah-Smith 2013 | English | + | + | + | + | + | + |
| Roschke 2000 | English | ? | ? | ? | ? | + | ? |
| Shen 2005 | English | + | ? | - | ? | + | ? |
| Sun 2013 | English | + | + | - | ? | - | ? |
| Wang 2014 | English | + | + | + | + | ? | - |
| Wenbin 2002 | English | + | ? | - | ? | + | ? |
| Whiting 2008 | English | ? | + | + | ? | - | ? |
| Xiujuan 1994 | English | ? | ? | - | ? | + | ? |
| Yeung 2011b | English | + | + | - | + | + | ? |
| Zhang 2003 | English | ? | ? | - | ? | + | ? |
| Zhang 2007 | English | ? | ? | - | ? | + | ? |
| Zhang 2009 | English | + | ? | + | + | + | ? |
| Zhang 2012 | English | + | ? | + | ? | + | + |
| Chau 2009 | English | + | ? | + | ? | - | ? |
| Chen 2016a | English | + | + | ? | + | - | + |
| Duan 1997 | English | ? | ? | ? | ? | ? | ? |
| Gosman‐Hedstrom 1998 | English | + | + | + | + | + | ? |
| Hopwood 2008 | English | + | + | - | + | + | ? |
| Hsieh 2007 | English | + | + | ? | + | - | ? |
| Hu 1993 | English | ? | ? | ? | ? | ? | ? |
| Johansson 1993 | English | + | + | ? | ? | ? | ? |
| Johansson 2001 | English | + | + | + | + | + | ? |
| Liu 2016 | English | + | + | ? | + | - | ? |
| Park 2005 | English | + | + | + | + | + | ? |
| Shen 2012b | English | + | + | + | ? | + | ? |
| Sze 2002 | English | + | + | ? | + | + | ? |
| Zhang 2015 | English | + | + | ? | + | + | + |
| Han 2008 | English | - | - | - | - | + | + |
| Kloster 1999 | English | ? | ? | + | + | + | + |
| Peng 2003 | English | + | ? | - | - | + | + |
| Xiong 2003 | English | ? | ? | - | - | + | + |
| Zhou 2000 | English | ? | ? | - | - | + | + |
| Garrow 2014 | English | + | + | - | + | - | ? |
| Han 2017 | English | ? | ? | - | - | + | ? |
| Zhang 2010 | English | ? | ? | - | - | + | ? |
| Ge 2016 | English | ? | ? | - | ? | + | ? |
| Lamontagne 1980 | English | ? | ? | + | + | + | ? |
| Leung 1991 | English | ? | ? | - | - | ? | ? |
| Cottraux 1983 | English | ? | ? | - | - | ? | ? |
| He 1997 | English | + | ? | + | + | + | ? |
| Steiner 1982 | English | ? | ? | + | + | ? | ? |
| Parker 1977b | English | ? | ? | + | + | + | ? |
| Parker 1977a | English | ? | ? | + | + | + | ? |
| Vibes 1977 | French | ? | ? | + | + | + | ? |
| Waite 1998 | English | ? | ? | + | + | + | ? |
| Martin 1981b | English | ? | ? | + | + | ? | ? |
| Gillams 1984 | English | + | + | + | + | + | ? |
| Martin 1981a | English | ? | ? | + | + | ? | ? |
| Wu 2007 | English | ? | ? | + | + | + | ? |
| Gilbey 1977 | English | ? | ? | + | + | ? | ? |
| White 1998 | English | + | + | + | + | ? | ? |
| Lacroix 1977 | French | ? | ? | + | + | ? | ? |
| Lagrue 1980 | French | - | - | + | + | - | ? |
| Clavel 1992 | English | ? | ? | + | + | ? | ? |
| Vandevenne 1985 | French | + | ? | + | + | ? | ? |
| Clavel 1992 +NG | English | ? | ? | + | + | ? | ? |
| Clavel 1985 | English | ? | ? | ? | ? | ? | ? |
| Circo 1985 | Italian | ? | ? | - | - | + | ? |
| Labadie 1983 | French | - | - | - | - | + | ? |
| Tian 1996 | English | ? | ? | - | - | ? | ? |
| White 2007 | English | + | + | - | - | - | ? |
| Zhang 2013 | English | + | + | + | + | - | ? |
| Wing 2010 | English | ? | ? | ? | ? | ? | ? |
| Bier 2002 | English | + | ? | + | + | - | ? |
| Chen 2016a | English | + | + | - | + | - | + |
| Jia 2006a | English | - | - | ? | ? | ? | ? |
| Liu 2000 | English | ? | ? | ? | ? | ? | ? |
| Liu 2004 | English | ? | ? | ? | ? | ? | ? |
| Chan 2012 | English | + | + | + | + | + | + |
| Xia 2016a | English | + | ? | ? | + | + | + |
| Cardini 1998 | English | + | + | + | + | + | + |
| Cardini 2005 | English | + | + | + | + | - | + |
| Guittier 2009 | English | + | + | + | + | + | + |
| Neri 2004 | English | + | + | + | + | - | + |
| Neri 2007 | English | ? | ? | ? | ? | + | + |
| Zhou 1999 | English | ? | ? | ? | ? | + | ? |
| Adib‐Hajbaghery 2013 | English | + | ? | + | - | ? | - |
| Agarwal 2000 | English | + | ? | ? | + | + | + |
| Agarwal 2002 | English | + | ? | + | + | + | + |
| Alkaissi 1999 | English | ? | ? | + | + | + | - |
| Alkaissi 2002 | English | ? | ? | + | + | + | + |
| Allen 1994 | English | ? | ? | + | ? | + | - |
| Amir 2007 | English | + | ? | ? | + | + | + |
| Andrzejowski 1996 | English | ? | ? | + | + | + | + |
| Arnberger 2007 | English | + | + | + | + | + | + |
| Barsoum 1990 | English | ? | ? | + | ? | + | - |
| Butkovic 2005 | English | ? | ? | + | + | + | - |
| Direkvand‐Moghadam 2013 | English | + | ? | ? | + | + | - |
| Duggal 1998 | English | + | ? | + | + | + | + |
| Dundee 1986 | English | ? | ? | + | + | + | ? |
| Dundee 1989 | English | ? | ? | ? | + | + | ? |
| Ebrahim Soltani 2010 | English | ? | ? | + | + | + | + |
| El‐Deeb 2011 | English | ? | ? | + | + | + | + |
| Ertas 2015 | English | + | + | + | + | + | - |
| Fassoulaki 1993 | English | ? | ? | + | + | - | - |
| Ferrara‐Love 1996 | English | - | ? | + | + | + | - |
| Frey 2009a | English | ? | ? | + | + | + | - |
| Frey 2009b | English | ? | ? | + | + | + | + |
| Gan 2004 | English | + | + | + | + | + | + |
| Gieron 1993 | German | ? | ? | + | - | + | + |
| Habib 2006 | English | ? | ? | + | + | + | - |
| Harmon 1999 | English | + | ? | + | + | - | + |
| Harmon 2000 | English | ? | ? | + | + | + | - |
| Ho 1990 | English | ? | ? | ? | ? | + | - |
| Ho 1996 | English | + | ? | + | + | + | + |
| Iqbal 2012 | English | ? | ? | + | + | + | + |
| Kim 2004 | English | ? | ? | + | + | + | + |
| Kim 2011 | English | ? | ? | + | + | + | + |
| Klein 2004 | English | + | ? | + | + | + | + |
| Koo 2013 | English | ? | ? | + | + | + | - |
| Lewis 1991 | English | ? | ? | + | + | + | - |
| Liu 2008 | English | + | ? | + | + | + | + |
| Majholm 2011 | English | + | + | + | + | + | + |
| Misra 2005 | English | + | ? | + | + | + | + |
| Nilsson 2015 | English | + | + | + | + | + | + |
| Ravi 2010 | English | + | ? | + | + | + | - |
| Rusy 2002 | English | + | ? | + | + | + | ? |
| Sadighha 2008 | English | - | - | + | + | + | - |
| Samad 2003 | English | + | ? | + | + | + | + |
| Schlager 1998 | English | ? | ? | + | ? | + | ? |
| Schultz 2003 | English | + | + | + | ? | - | - |
| Sharma 2007 | English | ? | ? | - | - | + | + |
| Shenkman 1999 | English | ? | ? | + | + | + | + |
| Streitberger 2004 | English | ? | + | + | + | + | + |
| Tavlan 1996 | English | ? | ? | ? | ? | + | ? |
| Turgut 2007 | English | ? | ? | + | + | + | + |
| Wang 2002 | English | + | ? | + | + | + | + |
| Wang 2010 | English | + | ? | + | + | + | + |
| White 2002 | English | + | ? | + | + | + | + |
| White 2012 | English | + | ? | + | + | + | + |
| Xu 2012 | English | + | + | + | + | + | + |
| Yang 1993 | English | ? | ? | ? | ? | + | - |
| Yentis 1992 | English | ? | ? | + | ? | + | - |
| Zárate 2001 | English | + | ? | + | + | + | + |
| Park 2009 | English | + | ? | + | + | - | + |
| Zhou 2005 | English | ? | ? | - | ? | + | - |
| Jedel 2011 | English | + | + | - | - | ? | + |
| Johansson 2013 | English | + | ? | - | ? | + | ? |
| Lim 2014 | English | + | + | ? | + | + | ? |
| Pastore 2011 | English | + | + | + | + | - | + |
| Wu 2017 | English | + | + | + | - | ? | + |
| Anastasi 2009 | English | + | + | + | + | + | + |
| Forbes 2005 | English | + | + | + | + | + | + |
| Lembo 2009 | English | + | + | + | + | + | + |
| Lowe 2000 | English | + | + | ? | ? | ? | + |
| Reynolds 2008 | English | + | + | - | - | + | - |
| Schneider 2006 | English | + | + | + | + | + | + |
| Fink 2001 | English | + | ? | + | + | + | + |
| Haslam 2001 | English | + | ? | - | - | - | - |
| Stener-Victorin 2004 | English | + | + | - | - | + | + |
| White 2012 | English | + | + | + | + | + | + |
| Witt 2006 | English | + | + | - | - | + | + |
| Aghamiri 2005 | Ottoman Turkish | + | ? | ? | ? | - | ? |
| Bazarganipour 2010 | English | + | ? | - | - | - | ? |
| Bu 2011 | English | ? | ? | - | - | + | ? |
| Charandabi 2011 | English | ? | ? | - | - | + | ? |
| Chen 2004 | English | + | + | - | - | - | ? |
| Chen 2010 | English | + | + | - | - | - | ? |
| Darabi 2010 | Persian | ? | ? | ? | ? | ? | ? |
| Helms 1987 | English | + | ? | - | - | + | ? |
| Kashefi 2010 | English | + | ? | ? | ? | ? | ? |
| Ma 2010 | English | + | + | + | + | + | ? |
| Ma 2013 | English | + | + | - | - | - | ? |
| Mirbagher-Ajorpaz 2011 | English | + | ? | ? | ? | + | ? |
| Shi 2011 | English | + | ? | - | - | + | ? |
| Smith 2010 | English | + | + | + | + | + | + |
| Sriprasert 2015 | English | + | + | - | - | - | ? |
| Wang 2009a | English | + | + | + | + | + | ? |
| Witt 2008 | English | + | + | - | - | + | ? |
| Wong 2010 | English | ? | ? | - | - | + | ? |
| Yeh 2013a | English | + | ? | ? | ? | - | ? |
| Zafari 2011 | English | ? | ? | - | - | + | - |
| Borup 2009 | English | + | + | - | - | + | ? |
| Calik 2014 | English | + | + | - | - | - | ? |
| Chung 2003 | English | + | + | ? | ? | + | ? |
| Dabiri 2014 | English | + | + | - | + | + | ? |
| Dong 2015 | English | + | ? | - | - | + | ? |
| Hamidzadeh 2012 | English | + | ? | - | + | + | ? |
| Hamlaci 2017 | English | + | + | - | - | + | ? |
| Hantoushzadeh 2007 | English | + | + | ? | + | + | ? |
| Hjelmstedt 2010 | English | + | + | ? | + | + | ? |
| Kashanian 2010 | English | + | + | ? | + | ? | ? |
| Kordi 2010 | Arabic | + | ? | - | - | ? | ? |
| Lee 2004 | English | + | ? | + | + | ? | ? |
| Ma 2011 | English | + | ? | - | ? | ? | ? |
| Mackenzie 2011 | English | + | + | - | + | ? | ? |
| Mafetoni 2016 | English | + | ? | + | + | ? | ? |
| Mafetoni 2016a | Portuguese | ? | + | + | + | - | ? |
| Mansouri 2018 | English | ? | + | ? | + | + | ? |
| Martensson 2008 | English | + | + | - | ? | + | ? |
| Nesheim 2003 | English | + | + | - | ? | - | ? |
| Ozgoli 2016 | English | + | - | - | - | + | ? |
| Qu 2007 | English | + | ? | - | ? | + | ? |
| Ramnero 2002 | English | + | + | ? | ? | + | ? |
| Salehian 2011 | Persian | + | + | - | ? | + | ? |
| Sehhatie-Shafaie 2013 | English | + | ? | + | ? | ? | + |
| Skilnand 2002 | English | + | + | + | ? | + | ? |
| Tjung 2008 | English | + | ? | - | + | ? | ? |
| Vixner 2014 | English | + | + | - | - | ? | + |
| Birch 1998 | English | ? | ? | + | + | + | ? |
| Cameron 2011 | English | + | + | + | + | + | + |
| Chou 2009 | English | + | ? | + | + | + | ? |
| Coan 1982 | English | + | - | - | - | + | ? |
| Fu 2009 | English | + | + | + | + | + | ? |
| He 2004 | English | + | ? | + | + | + | ? |
| He 2005 | English | ? | ? | - | - | + | ? |
| Ilbuldu 2004 | English | ? | ? | - | - | - | ? |
| Irnich 2001 | English | + | ? | - | - | + | ? |
| Irnich 2002 | English | + | + | + | + | + | ? |
| Itoh 2007 | English | + | ? | - | - | - | ? |
| Kwak 2012 | English | + | ? | - | ? | + | + |
| Liang 2011 | English | + | + | + | + | + | ? |
| Nabeta 2002 | English | + | ? | + | + | + | ? |
| Petrie 1983 | English | ? | ? | - | - | + | ? |
| Petrie 1986 | English | ? | ? | - | - | + | ? |
| Sahin 2010 | English | - | - | + | + | + | ? |
| Seidel 2002 | German | + | ? | + | + | + | ? |
| Sun 2010 | English | ? | ? | ? | ? | + | ? |
| Thomas 1991 | English | ? | ? | - | - | + | ? |
| Tough 2010 | English | + | + | ? | ? | + | ? |
| Tsai 2010 | English | + | ? | + | + | + | ? |
| Vas 2006 | English | + | ? | + | + | - | ? |
| White 2000 | English | ? | ? | - | - | ? | ? |
| White 2004 | English | + | ? | - | - | + | ? |
| Witt 2006 | English | + | + | - | - | - | ? |
| Flachskampf 2007 | English | ? | + | + | + | + | + |
| Kim 2012 | English | ? | + | + | + | - | + |
| Macklin 2006 | English | + | ? | + | + | + | + |
| Yin 2007 | English | + | ? | - | - | - | + |
| Chou 2009 | English | ? | ? | ? | ? | + | ? |
| Naeser 1992 | English | ? | ? | + | ? | ? | ? |
| Assefi 2005 | English | + | + | + | + | + | + |
| Deluze 1992 | English | + | + | + | + | - | + |
| Harris 2005 | English | + | + | + | + | + | + |
| Harris 2008 | English | + | + | + | + | + | ? |
| Harris 2009 | English | + | + | + | + | + | ? |
| Itoh 2010 | English | + | + | - | + | - | ? |
| Martin 2006 | English | ? | + | + | + | + | ? |
| Targino 2008 | English | + | - | - | + | + | + |
| Aigner 1999 | German | ? | ? | + | ? | + | ? |
| Chung 2016 | English | + | + | - | - | + | + |
| Hadianfard 2015 | English | + | - | - | ? | + | ? |
| Kumnerddee 2010 | English | + | ? | - | ? | + | ? |
| Weinstein 2003 | English | ? | ? | + | + | - | - |
| Yang 2009 | English | + | ? | - | - | + | + |
| Yao 2012 | English | + | ? | + | + | + | ? |
| Allam 2008 | English | + | + | + | + | + | + |
| Chan 2009 | English | ? | ? | - | - | + | + |
| Wong 2002 | English | + | + | - | - | + | + |
| Wong 2007 | English | + | + | + | + | + | + |
| Wong 2010a | English | + | + | + | + | + | + |
| Wong 2010b | English | + | + | + | + | + | + |
| Haker 1990a | English | + | ? | + | ? | + | ? |
| Haker 1990b | English | + | ? | + | - | + | ? |
| Molsberger 1994 | English | + | ? | + | + | + | ? |
| Che‐Yi 2005 | English | + | ? | ? | + | + | ? |
| Cho 2004 | English | - | - | - | ? | + | ? |
| Gao 2002 | English | ? | ? | - | ? | ? | ? |
| Hsu 2009 | English | + | ? | ? | + | - | ? |
| Jedras 2003 | English | ? | ? | ? | ? | ? | ? |
| Lin 2011 | English | + | ? | - | ? | + | - |
| Ma 2004 | English | - | - | - | ? | + | ? |
| Qiu 2012 | English | + | ? | - | ? | + | ? |
| Shariati 2012 | English | ? | ? | - | + | - | ? |
| Su 2009 | English | ? | ? | ? | ? | ? | ? |
| Tsay 2003a | English | ? | ? | ? | + | ? | ? |
| Tsay 2004a | English | ? | ? | ? | ? | ? | ? |
| Tsay 2004b | English | ? | ? | - | ? | ? | ? |
| Kim 2012 | English | + | + | - | + | ? | - |
| Tsay 2004 | English | ? | ? | - | + | + | + |
| Arab 2016 | English | ? | ? | - | + | - | + |
| Shariati 2012 | English | ? | ? | - | ? | ? | + |
| Tsay 2003a | English | ? | ? | - | + | - | + |
| Zou 2015 | English | + | ? | + | + | - | + |
| SIESTA 2017 | English | + | + | - | + | + | - |
| Sahin 2015 | English | + | ? | + | + | + | ? |
| Lee 2009 | English | + | ? | + | + | + | ? |
| Lee 2008 | English | + | ? | + | + | + | + |
| Kucuk 2015 | English | ? | ? | - | - | + | ? |
| Leskinen 2002 | English | ? | ? | ? | ? | + | ? |
| Radmayr 2001 | English | + | ? | ? | ? | ? | ? |
| Paris 1983 | English | - | - | - | - | + | - |
| Sator-Katzenschlager 2006 | English | + | ? | + | + | + | + |
| Gejervall 2005 | English | + | ? | + | + | + | + |
| Humaidan 2004 | English | + | + | ? | ? | + | + |
| Stener-Victorin 1999 | English | + | + | ? | ? | + | + |
| Stener-Victorin 2003 | English | + | + | ? | ? | ? | + |
| Ajori 2013 | English | + | + | + | + | + | + |
| Alsharnoubi 2015 | English | - | - | + | ? | ? | ? |
| Andersen 2013 | English | + | + | + | + | + | ? |
| Asher 2009 | English | + | + | + | + | + | ? |
| Gaudernack 2006 | English | + | + | + | ? | + | ? |
| Gaudet 2008 | English | + | + | + | + | + | ? |
| Gregson 2015 | English | + | + | + | + | + | + |
| Gribel 2011 | English | + | + | + | + | + | ? |
| Harper 2006 | English | + | + | + | ? | + | ? |
| Mackenzie 2011 | English | + | + | + | + | + | + |
| Martinez 2004 | English | ? | ? | - | ? | + | + |
| Modlock 2010 | English | + | + | ? | + | + | + |
| Mollart 2016 | English | + | + | + | + | + | ? |
| Neri 2014 | English | - | ? | + | ? | + | ? |
| Rabl 2001 | English | + | + | + | - | - | ? |
| Romer 2000 | German | + | ? | + | + | + | ? |
| Selmer-Olsen 2007 | English | + | + | + | ? | - | - |
| Smith 2008 | English | + | + | + | + | + | + |
| Torkzahrani 2015 | English | ? | ? | ? | ? | - | ? |
| Torkzahrani 2016 | English | + | ? | + | + | + | - |
| Tremeau 1992 | French | ? | ? | ? | + | ? | ? |
| Duggal 1998 | English | + | ? | + | + | ? | ? |
| El-Deeb 2011a | English | ? | ? | ? | + | ? | ? |
| Habib 2006 | English | + | ? | + | + | + | ? |
| Harmon 2000 | English | ? | ? | ? | + | ? | ? |
| Ho 1996 | English | + | ? | + | + | + | ? |
| Ho 2006 | English | ? | ? | + | + | + | ? |
| Levin 2019 | English | + | ? | ? | ? | + | ? |
| Noroozinia 2013 | English | ? | ? | ? | ? | ? | ? |
| Stein 1997 | English | ? | ? | + | + | + | ? |
| Direkvand-Moghadam 2013 | English | + | ? | - | + | + | ? |
| Birnbach 1993 | English | ? | ? | ? | ? | ? | ? |
| Lim 2001a | English | ? | ? | ? | ? | ? | ? |
| Lim 2001b | English | ? | ? | ? | ? | ? | ? |
| Lee 2007 | Korea | + | ? | ? | ? | - | ? |
| Youn 2008 | Korea | + | ? | - | - | - | ? |
| Cho 1977 | Korea | ? | ? | - | ? | + | - |

**References**

1. Armour M, Ee CC, Hao J, Wilson TM, Yao SS, Smith CA. Acupuncture and acupressure for premenstrual syndrome. Cochrane Database of Systematic Reviews. 2018(8).

2. Bath PM, Lee HS, Everton LF. Swallowing therapy for dysphagia in acute and subacute stroke. Cochrane Database of Systematic Reviews. 2018(10).

3. Cao H, Yang G, Wang Y, Liu JP, Smith CA, Luo H, et al. Complementary therapies for acne vulgaris. Cochrane Database of Systematic Reviews. 2015(1).

4. Cheng K, Law A, Guo M, Wieland LS, Shen X, Lao L. Acupuncture for acute hordeolum. Cochrane Database of Systematic Reviews. 2017(2).

5. Cheuk DKL, Wong V. Acupuncture for epilepsy. Cochrane Database of Systematic Reviews. 2014(5).

6. Cheuk DKL, Wong V, Chen WX. Acupuncture for autism spectrum disorders (ASD). Cochrane Database of Systematic Reviews. 2011(9).

7. Choi GH, Wieland LS, Lee H, Sim H, Lee MS, Shin BC. Acupuncture and related interventions for the treatment of symptoms associated with carpal tunnel syndrome. Cochrane Database of Systematic Reviews. 2018(12).

8. Coyle ME, Smith CA, Peat B. Cephalic version by moxibustion for breech presentation. Cochrane Database of Systematic Reviews. 2012(5).

9. Crepinsek MA, Taylor EA, Michener K, Stewart F. Interventions for preventing mastitis after childbirth. The Cochrane database of systematic reviews. 2020;9(9):Cd007239.

10. Cui Y, Wang Y, Liu Z. Acupuncture for restless legs syndrome. Cochrane Database of Systematic Reviews. 2008(4).

11. Deare JC, Zheng Z, Xue CCL, Liu JP, Shang J, Scott SW, et al. Acupuncture for treating fibromyalgia. Cochrane Database of Systematic Reviews. 2013(5).

12. Franco JVA, Turk T, Jung JH, Xiao YT, Iakhno S, Garrote V, et al. Non‐pharmacological interventions for treating chronic prostatitis/chronic pelvic pain syndrome. Cochrane Database of Systematic Reviews. 2018(5).

13. Green S, Buchbinder R, Barnsley L, Hall S, White M, Smidt N, et al. Acupuncture for lateral elbow pain. Cochrane Database of Systematic Reviews. 2002(1).

14. Griffiths JD, Gyte GM, Popham PA, Williams K, Paranjothy S, Broughton HK, et al. Interventions for preventing nausea and vomiting in women undergoing regional anaesthesia for caesarean section. The Cochrane database of systematic reviews. 2021;5(5):Cd007579.

15. Huang T, Shu X, Huang YS, Cheuk DKL. Complementary and miscellaneous interventions for nocturnal enuresis in children. Cochrane Database of Systematic Reviews. 2011(12).

16. Ju ZY, Wang K, Cui HS, Yao Y, Liu SM, Zhou J, et al. Acupuncture for neuropathic pain in adults. Cochrane Database of Systematic Reviews. 2017(12).

17. Kim KH, Lee MS, Choi TY, Kim TH. Acupuncture for symptomatic gastroparesis. Cochrane Database of Systematic Reviews. 2018(12).

18. Kim KH, Lee MS, Kim TH, Kang JW, Choi TY, Lee JD. Acupuncture and related interventions for symptoms of chronic kidney disease. Cochrane Database of Systematic Reviews. 2016(6).

19. Kim TH, Lee MS, Kim KH, Kang JW, Choi TY, Ernst E. Acupuncture for treating acute ankle sprains in adults. Cochrane Database of Systematic Reviews. 2014(6).

20. Kong DZ, Liang N, Yang GL, Zhang Z, Liu Y, Yang Y, et al. Acupuncture for chronic hepatitis B. Cochrane Database of Systematic Reviews. 2019(8).

21. Kwan I, Wang R, Pearce E, Bhattacharya S. Pain relief for women undergoing oocyte retrieval for assisted reproduction. Cochrane Database of Systematic Reviews. 2018(5).

22. Lan L, Zeng F, Liu GJ, Ying L, Wu X, Liu M, et al. Acupuncture for functional dyspepsia. Cochrane Database of Systematic Reviews. 2014(10).

23. Lee A, Chan SKC, Fan LTY. Stimulation of the wrist acupuncture point PC6 for preventing postoperative nausea and vomiting. Cochrane Database of Systematic Reviews. 2015(11).

24. Lim CED, Ng RWC, Cheng NCL, Zhang GS, Chen H. Acupuncture for polycystic ovarian syndrome. Cochrane Database of Systematic Reviews. 2019(7).

25. Manheimer E, Cheng K, Wieland LS, Min LS, Shen X, Berman BM, et al. Acupuncture for treatment of irritable bowel syndrome. Cochrane Database of Systematic Reviews. 2012(5).

26. Manheimer E, Cheng K, Wieland LS, Shen X, Lao L, Guo M, et al. Acupuncture for hip osteoarthritis. Cochrane Database of Systematic Reviews. 2018(5).

27. Mitchell C, Bowen A, Tyson S, Butterfint Z, Conroy P. Interventions for dysarthria due to stroke and other adult‐acquired, non‐progressive brain injury. Cochrane Database of Systematic Reviews. 2017(1).

28. Natale P, Ruospo M, Saglimbene VM, Palmer SC, Strippoli GFM. Interventions for improving sleep quality in people with chronic kidney disease. Cochrane Database of Systematic Reviews. 2019(5).

29. Shen X, Xia J, Adams CE. Acupuncture for schizophrenia. Cochrane Database of Systematic Reviews. 2014(10).

30. Sinopoulou V, Gordon M, Akobeng AK, Gasparetto M, Sammaan M, Vasiliou J, et al. Interventions for the management of abdominal pain in Crohn's disease and inflammatory bowel disease. The Cochrane database of systematic reviews. 2021;11:Cd013531.

31. Smith CA, Armour M, Dahlen HG. Acupuncture or acupressure for induction of labour. Cochrane Database of Systematic Reviews. 2017(10).

32. Smith CA, Armour M, Lee MS, Wang LQ, Hay PJ. Acupuncture for depression. Cochrane Database of Systematic Reviews. 2018(3).

33. Smith CA, Armour M, Zhu X, Li X, Lu ZY, Song J. Acupuncture for dysmenorrhoea. Cochrane Database of Systematic Reviews. 2016(4).

34. Smith CA, Collins CT, Levett KM, Armour M, Dahlen HG, Tan AL, et al. Acupuncture or acupressure for pain management during labour. Cochrane Database of Systematic Reviews. 2020(2).

35. Teixeira LJ, Valbuza JS, Prado GF. Physical therapy for Bell's palsy (idiopathic facial paralysis). Cochrane Database of Systematic Reviews. 2011(12).

36. Thomas LH, Coupe J, Cross LD, Tan AL, Watkins CL. Interventions for treating urinary incontinence after stroke in adults. Cochrane Database of Systematic Reviews. 2019(2).

37. Trinh K, Graham N, Irnich D, Cameron ID, Forget M. Acupuncture for neck disorders. Cochrane Database of Systematic Reviews. 2016(11).

38. Wang Y, Zhishun L, Peng W, Zhao J, Liu B. Acupuncture for stress urinary incontinence in adults. Cochrane Database of Systematic Reviews. 2013(7).

39. White AR, Rampes H, Liu JP, Stead LF, Campbell J. Acupuncture and related interventions for smoking cessation. Cochrane Database of Systematic Reviews. 2014(1).

40. Wong V, Cheuk DKL, Lee S, Chu V. Acupuncture for acute management and rehabilitation of traumatic brain injury. Cochrane Database of Systematic Reviews. 2013(3).

41. Xie Y, Wang L, He J, Wu T. Acupuncture for dysphagia in acute stroke. Cochrane Database of Systematic Reviews. 2008(3).

42. Xu M, Li D, Zhang S. Acupuncture for acute stroke. Cochrane Database of Systematic Reviews. 2018(3).

43. Yang A, Wu HM, Tang JL, Xu L, Yang M, Liu GJ. Acupuncture for stroke rehabilitation. Cochrane Database of Systematic Reviews. 2016(8).

44. Yang J, Chen J, Yang M, Yu S, Ying L, Liu GJ, et al. Acupuncture for hypertension. Cochrane Database of Systematic Reviews. 2018(11).

45. Zhu X, Proctor M, Bensoussan A, Wu E, Smith CA. Chinese herbal medicine for primary dysmenorrhoea. Cochrane Database of Systematic Reviews. 2008(2).

46. Abdel Hay R, Shalaby K, Zaher H, Hafez V, Chi CC, Dimitri S, et al. Interventions for acne scars. Cochrane Database of Systematic Reviews. 2016(4).

47. Boelig RC, Barton SJ, Saccone G, Kelly AJ, Edwards SJ, Berghella V. Interventions for treating hyperemesis gravidarum. Cochrane Database of Systematic Reviews. 2016(5).

48. Casimiro L, Barnsley L, Brosseau L, Milne S, Welch V, Tugwell P, et al. Acupuncture and electroacupuncture for the treatment of rheumatoid arthritis. Cochrane Database of Systematic Reviews. 2005(4).

49. Cheong YC, Dix S, Hung Yu Ng E, Ledger WL, Farquhar C. Acupuncture and assisted reproductive technology. Cochrane Database of Systematic Reviews. 2013(7).

50. David JA, Sankarapandian V, Christopher PRH, Chatterjee A, Macaden AS. Injected corticosteroids for treating plantar heel pain in adults. Cochrane Database of Systematic Reviews. 2017(6).

51. Dennis CL, Dowswell T. Interventions (other than pharmacological, psychosocial or psychological) for treating antenatal depression. Cochrane Database of Systematic Reviews. 2013(7).

52. Dodin S, Blanchet C, Marc I, Ernst E, Wu T, Vaillancourt C, et al. Acupuncture for menopausal hot flushes. Cochrane Database of Systematic Reviews. 2013(7).

53. Eachempati P, Kumbargere Nagraj S, Kiran Kumar Krishanappa S, George RP, Soe HHK, Karanth L. Management of gag reflex for patients undergoing dental treatment. Cochrane Database of Systematic Reviews. 2019(11).

54. Every‐Palmer S, Newton‐Howes G, Clarke MJ. Pharmacological treatment for antipsychotic‐related constipation. Cochrane Database of Systematic Reviews. 2017(1).

55. Farrell D, Artom M, Czuber‐Dochan W, Jelsness‐Jørgensen LP, Norton C, Savage E. Interventions for fatigue in inflammatory bowel disease. Cochrane Database of Systematic Reviews. 2020(4).

56. Furlan AD, Giraldo M, Baskwill A, Irvin E, Imamura M. Massage for low‐back pain. Cochrane Database of Systematic Reviews. 2015(9).

57. Griffiths JD, Gyte GML, Paranjothy S, Brown HC, Broughton HK, Thomas J. Interventions for preventing nausea and vomiting in women undergoing regional anaesthesia for caesarean section. Cochrane Database of Systematic Reviews. 2012(9).

58. Gross A, Kay TM, Paquin JP, Blanchette S, Lalonde P, Christie T, et al. Exercises for mechanical neck disorders. Cochrane Database of Systematic Reviews. 2015(1).

59. Gross A, Langevin P, Burnie SJ, Bédard‐Brochu MS, Empey B, Dugas E, et al. Manipulation and mobilisation for neck pain contrasted against an inactive control or another active treatment. Cochrane Database of Systematic Reviews. 2015(9).

60. Karjalainen TV, Silagy M, O'Bryan E, Johnston RV, Cyril S, Buchbinder R. Autologous blood and platelet‐rich plasma injection therapy for lateral elbow pain. Cochrane Database of Systematic Reviews. 2021(9).

61. Kumbargere Nagraj S, George RP, Shetty N, Levenson D, Ferraiolo DM, Shrestha A. Interventions for managing taste disturbances. Cochrane Database of Systematic Reviews. 2017(12).

62. Liddle SD, Pennick V. Interventions for preventing and treating low‐back and pelvic pain during pregnancy. Cochrane Database of Systematic Reviews. 2015(9).

63. Linde K, Allais G, Brinkhaus B, Fei Y, Mehring M, Shin BC, et al. Acupuncture for the prevention of tension‐type headache. Cochrane Database of Systematic Reviews. 2016(4).

64. Linde K, Allais G, Brinkhaus B, Fei Y, Mehring M, Vertosick EA, et al. Acupuncture for the prevention of episodic migraine. Cochrane Database of Systematic Reviews. 2016(6).

65. Manheimer E, Cheng K, Linde K, Lao L, Yoo J, Wieland S, et al. Acupuncture for peripheral joint osteoarthritis. Cochrane Database of Systematic Reviews. 2010(1).

66. Manyande A, Cyna AM, Yip P, Chooi C, Middleton P. Non‐pharmacological interventions for assisting the induction of anaesthesia in children. Cochrane Database of Systematic Reviews. 2015(7).

67. Matthews A, Haas DM, O'Mathúna DP, Dowswell T. Interventions for nausea and vomiting in early pregnancy. Cochrane Database of Systematic Reviews. 2015(9).

68. McCarney RW, Brinkhaus B, Lasserson TJ, Linde K. Acupuncture for chronic asthma. Cochrane Database of Systematic Reviews. 2003(3).

69. Middleton P, Shepherd E, Flenady V, McBain RD, Crowther CA. Planned early birth versus expectant management (waiting) for prelabour rupture of membranes at term (37 weeks or more). Cochrane Database of Systematic Reviews. 2017(1).

70. Moraes VY, Lenza M, Tamaoki MJ, Faloppa F, Belloti JC. Platelet‐rich therapies for musculoskeletal soft tissue injuries. Cochrane Database of Systematic Reviews. 2014(4).

71. Natale P, Palmer SC, Ruospo M, Saglimbene VM, Rabindranath KS, Strippoli GFM. Psychosocial interventions for preventing and treating depression in dialysis patients. Cochrane Database of Systematic Reviews. 2019(12).

72. O'Connor D, Marshall SC, Massy‐Westropp N, Pitt V. Non‐surgical treatment (other than steroid injection) for carpal tunnel syndrome. Cochrane Database of Systematic Reviews. 2003(1).

73. Patel KC, Gross A, Graham N, Goldsmith CH, Ezzo J, Morien A, et al. Massage for mechanical neck disorders. Cochrane Database of Systematic Reviews. 2012(9).

74. Phupong V, Hanprasertpong T. Interventions for heartburn in pregnancy. Cochrane Database of Systematic Reviews. 2015(9).

75. Saensak S, Vutyavanich T, Somboonporn W, Srisurapanont M. Relaxation for perimenopausal and postmenopausal symptoms. Cochrane Database of Systematic Reviews. 2014(7).

76. Smith CA, Collins CT, Cyna AM, Crowther CA. Complementary and alternative therapies for pain management in labour. Cochrane Database of Systematic Reviews. 2006(4).

77. Stevens B, Yamada J, Ohlsson A, Haliburton S, Shorkey A. Sucrose for analgesia in newborn infants undergoing painful procedures. Cochrane Database of Systematic Reviews. 2016(7).

78. Surace SJ, Deitch J, Johnston RV, Buchbinder R. Shock wave therapy for rotator cuff disease with or without calcification. Cochrane Database of Systematic Reviews. 2020(3).

79. van Durme CM, Wechalekar MD, Buchbinder R, Schlesinger N, van der Heijde D, Landewé RB. Non-steroidal anti-inflammatory drugs for acute gout. The Cochrane database of systematic reviews. 2014(9):Cd010120.

80. Verhagen AP, Scholten‐Peeters G, van Wijngaarden S, de Bie R, Bierma‐Zeinstra SMA. Conservative treatments for whiplash. Cochrane Database of Systematic Reviews. 2007(2).

81. Vogel JP, Osoti AO, Kelly AJ, Livio S, Norman JE, Alfirevic Z. Pharmacological and mechanical interventions for labour induction in outpatient settings. Cochrane Database of Systematic Reviews. 2017(9).

82. Zakarija-Grkovic I, Stewart F. Treatments for breast engorgement during lactation. Cochrane Database of Systematic Reviews. 2020(9).

83. Zhu X, Hamilton KD, McNicol ED. Acupuncture for pain in endometriosis. Cochrane Database of Systematic Reviews. 2011(9).

84. Zimpel SA, Torloni MR, Porfírio GJM, Flumignan RLG, da Silva EMK. Complementary and alternative therapies for post‐caesarean pain. Cochrane Database of Systematic Reviews. 2020(9).
